# Identifying pastoral and plant products in local and imported pottery in Early Bronze Age southeastern Arabia

**DOI:** 10.1371/journal.pone.0324661

**Published:** 2025-06-11

**Authors:** Akshyeta Suryanarayan, Sophie Méry, Jennifer Swerida, Michele Degli Esposti, Eli Dollarhide, Stephanie Döpper, Khaled A. Douglas, Daniel Eddisford, Nasser S. Al-Jahwari, Arnaud Mazuy, Núria Moraleda-Cibrián, Michel de Vreeze, Joan Villanueva, Cameron A. Petrie, Martine Regert

**Affiliations:** 1 Faculty of Asian and Middle Eastern Studies, University of Oxford, Oxford, United Kingdom; 2 McDonald Institute for Archaeological Research, University of Cambridge, Cambridge, United Kingdom; 3 Université Côte d’Azur, CNRS, CEPAM, France; 4 CNRS, UMR 7041, Archéologies et sciences de l’Antiquité (ArScAn), Nanterre, France; 5 Faculty of Archaeology, Leiden University, Netherlands; 6 Institute of Mediterranean and Oriental Cultures, Polish Academy of Sciences, Warsaw, Poland; 7 New York University Abu Dhabi, Saadiyat Island, Abu Dhabi, UAE; 8 Heidelberg Center for Cultural Heritage, Heidelberg University, Germany; 9 Archaeology Department, Sultan Qaboos University, Sultanate of Oman; 10 Department of Archaeology, Durham University, United Kingdom; 11 Institute of Environmental Science and Technology (ICTA-UAB), Autonomous University of Barcelona, Barcelona, Spain; Banaras Hindu University, INDIA

## Abstract

The origins of ceramic technology in the Oman Peninsula have a unique history in the context of ancient West Asia. Local pottery production in northern Oman and the United Arab Emirates is not documented until the early to mid-third millennium BC during the Early Bronze Age. This period was characterised by increasing sedentism and the expansion of long-distance exchange networks that operated across the Persian Gulf between Arabia, Mesopotamia, Iran and South Asia, including the exchange of ceramic vessels. In order to explore the links between ceramic technology and type, subsistence practices and sedentism as ceramic production was adopted in the region, we analysed the lipid content of Early Bronze Age pottery (n = 179) in southeastern Arabia from inland and coastal sites. The ceramic assemblage examined includes pottery produced locally at the site level as well as vessels that are distributed regionally. The contents of imported pottery from Mesopotamia and the Indus Civilisation from inland and coastal sites were also studied to determine the organic products that may have been transported as part of long-distance exchange. The results reveal the presence of pastoral products, such as meat and dairy products, in some of the earliest vessels produced in southeastern Arabia, as well as imported Mesopotamian vessels. Plant products are detected in a small minority of vessels in locally-produced and imported vessels, such as Fine Red Omani vessels and Black-Slipped Jars from the Indus Civilisation. Such an investigation demonstrates the importance of using biomolecular methods to study dietary practices and vessel use in southeastern Arabia on a larger scale.

## 1. Introduction

**Fig 1:**
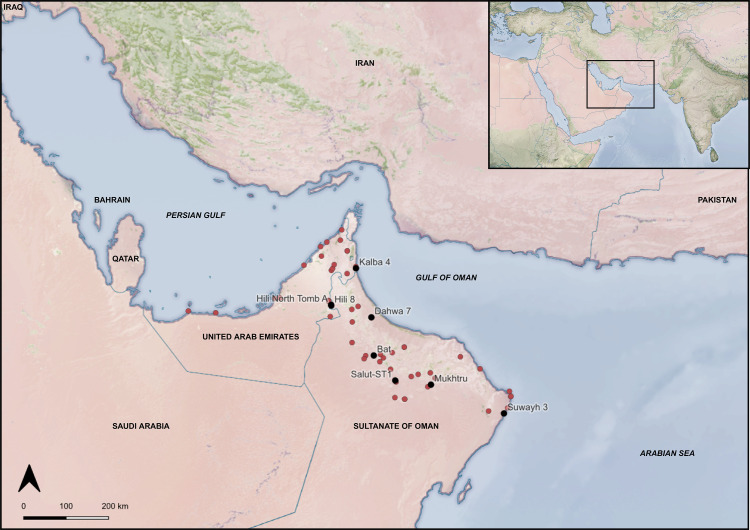
Map of southeastern Arabia with Early Bronze Age archaeological sites in filled red circles and study sites in filled black circles. Basemap: NASA Visible Earth [21].

Pottery technology was adopted in different parts of the world at different times through complex processes of invention or innovation and cultural transmission [1–5]. In ancient West Asia, pottery, farming and sedentism developed independently of each other and at different times between the ninth to the seventh millennia BC [6–9]. Southeastern Arabia (modern-day northern Sultanate of Oman and the United Arab Emirates) is a region that has traditionally been excluded from larger narratives about the development of social complexity, and it followed a unique trajectory in the adoption of pottery, sedentism and agriculture. Pottery technology was adopted at a significant scale in southeastern Arabia at a relatively fast pace only during the Early Bronze Age (EBA), particularly during the Umm an-Nar period as known in Arabian archaeology (c. 2800–2000 BC). This period is characterised by many social changes, including the growth of sedentary settlements [10], changes in funerary practices[11], the adoption of oasis agriculture [12], and the expansion of long-distance exchange networks between regions around the Persian Gulf and Arabian Sea [13,14]. Early locally-produced pottery in southeastern Arabia appears to be influenced by ceramic technologies from the Indo-Iranian borderlands [15–20]. Once adopted, domestic pottery was produced at individual sites; a finer variety was produced and distributed at a wider regional scale; and imported pottery from Mesopotamia, Makran, Sistan, and the Indus Civilisation was circulated and found at settlements and in tombs in southeastern Arabia. Our study examines these three different categories of pottery (local, regionally distributed, and imported, n = 179) from eight sites in southeastern Arabia using lipid residue analysis in order to examine the uses of vessel technology as it was more widely adopted in the region (Fig 1).

Lipid residue analysis of pottery has so far been applied in limited archaeological contexts in southeastern Arabia [see 22,23]. Lipids such as fats, oils, and waxes have relatively high potential for preservation in archaeological contexts, and provide a means to directly identify the contents of ancient vessels [24–26]. Preservation of other types of bioarchaeological remains in southeastern Arabia, such as macrobotanical [12], faunal [27,28], and human remains [29–31] is variable, which limits information about ancient human dietary practices. Given the success of initial lipid residue studies in southeastern Arabia [22,23], it is timely to study pottery from a larger number of sites to investigate broader patterns of subsistence practices and the relationship between pottery and organic substances in the region. Such an approach also lends a deeper understanding of the use of pottery and why it may have been adopted; asking questions beyond provenance determination, technological and shape determination aspects of pottery [31–32]. Importantly, the contents of imported vessels in southeastern Arabia, such as pottery from Mesopotamia and Black-Slipped Jars from the Indus Civilisation (specifically the lower Indus plain), can also be directly detected and characterised. Given the limited archaeological evidence of the ‘invisible’ trade in organic products that likely played a major role in ancient long-distance exchange networks, such evidence adds a crucial new element to our understanding of the complex and intertwining networks of production and exchange that existed both within southeastern Arabia as well as between areas along the Persian Gulf littoral during the EBA.

### 1.1 The adoption of pottery during the EBA in southeastern Arabia

Pottery from Mesopotamia (Ubaid period, c. 5000−4500 BC, Periods 2 and 2/3;) was in circulation in southeastern Arabia from the mid-sixth millennium BC, found at coastal midden sites in the UAE [33–35]. Evidence for settlements dating to the fourth millennium BC are limited, likely influenced by climatic shifts [36,37]. In late fourth-early third millennium BC, during the Hafit period (c. 3200−2800 BC) [38], pottery vessels with clear typological links to Jemdet Nasr and Early Dynastic shapes that largely originated in southern Mesopotamia [31] are found at beehive-shaped collective tombs across southeastern Arabia [39]. The available evidence suggests that imported pottery, and possibly their contents, may have been votive offerings in funerary practices at the time. There are a very limited number of known or excavated Hafit settlements. Hafit-period communities in southeastern Arabia appear to not have used fired pottery in domestic settings despite being in contact with ceramic-producing groups and having knowledge of complex pyrotechnologies utilised in copper production and softstone bead production [15,20].

**Fig 2:**
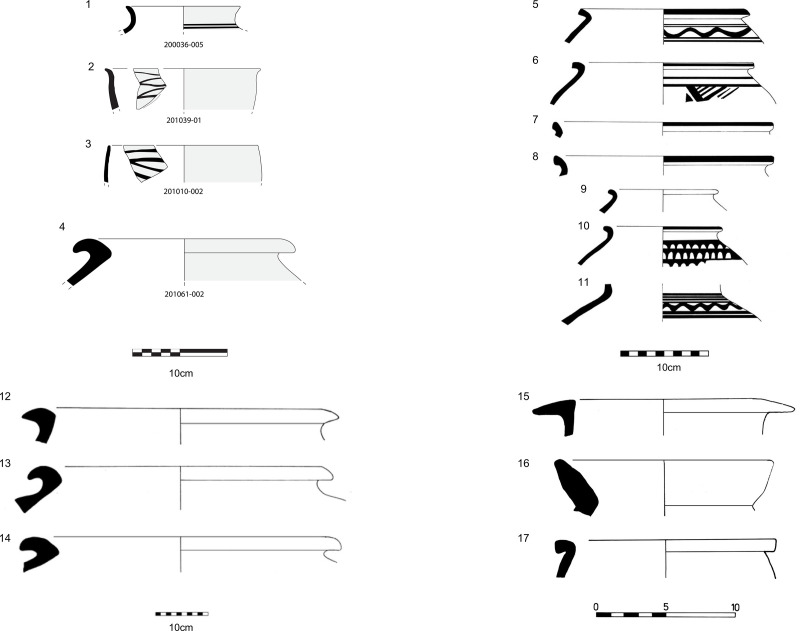
Examples of local Umm an-Nar Sandy/Domestic ware fragments (1-3) and Indus Black-Slipped Jar fragment from Bat (4), Fine Red Omani ware/Fine Red Ware (5-11) and Indus Black-Slipped Jar fragments from Kalba 4 (12-14), and Mesopotamian pottery from Hili 8 (15-17). Credits: Pottery from Bat drawn by Reilly Jensen and Jennifer Swerida, pottery from Kalba drawn by Daniel Eddisford, and pottery from Hili 8 drawn by Hélène David-Cuny.

The earliest-known locally-produced pottery in southeastern Arabia dates to around c. 3000 BC at the settlement of Hili 8 and appears alongside imported southern Mesopotamian pottery [40: 52] (Fig 2). This early pottery is fine, black-painted and red-slipped, and draws on the stylistic traditions of the Indo-Iranian region. It has been suggested that the rapid appearance of these ceramics and high technical knowledge required to create them was associated with the movement of craftspeople [18,19]; which is supported by geochemical studies showing that at least some of these vessels were produced on the Dasht plain in Pakistani Makran [41]. The early third millennium BC dates of Hili 8’s sequence have been questioned [42: 66–67], but, despite its limited publication, it is one of the few settlement sites with an established EBA ceramic chronology in the region (S1 File). Apart from Hili 8, evidence of imported Mesopotamian Jemdet Nasr and Early Dynastic I-II pottery along with locally-produced, crude copies of Mesopotamian wares, black-on-red sherds and crucibles during the later Hafit and early Umm an-Nar period (c. 2900–2600 BC) are also found at the site of Bat [20: 189–193]. The evidence from Hili 8 and Bat thus suggests that the adoption of ceramic technology in Oman was a complex process that likely involved influences from the Indo-Iranian region as well as Mesopotamia [19,20,43].

By *c*. 2500 BC during the Umm an-Nar period (c. 2800−2000 BC), domestic pottery with a sandy paste and geometric decorations painted in black-on-red slip, which we hereafter refer to as ‘locally-produced’ pottery (Fig 2), began to be produced within individual settlements [17]. A finer variety of homogenously produced, red-slipped black-painted ware was distributed at tombs and settlements across southeastern Arabia [17], referred to as ‘regional pottery’ in this paper. By this period, settlements with mudbrick constructions, many with stone or mudbrick ‘towers’, with associated wells, usually within wadis or alluvial plains, were found across the region indicating a wider shift toward sedentism [10,14: 98–103]. There was also a change in funerary practices, where large communal stone tombs were built for the interment of many individuals over several generations [11]. Combined with cattle, sheep/goat husbandry that was practiced since the Neolithic, ~ c. 6500−5000 BC [44,45], the growth of oasis agrosystems is suggested at inland sites [12,46,47], while fishing and the gathering of shellfish is hypothesised to have continued to play an important role in the subsistence economy at coastal settlements [28: 474].

### 1.2 Inland and coastal subsistence in southeastern Arabia in the Early Bronze Age

Archaeobotanical researchsuggests the consumption of a range of C_3_ plants including wheat, emmer, two-rowed barley, six-rowed barley, oat, pea, melon, and date palm during the EBA in southeastern Arabia, reported at the sites of Hili 8, Bat and Tell Abraq [46–50]. Populations at inland settlements are hypothesised to have relied more on agricultural products grown in date-palm gardens [12,30], as well as domestic animal husbandry [28]. There are only a few inland EBA sites for which detailed bioarchaeological information exists, particularly due to poor preservation [27,28] and inconsistent sampling techniques. For example, at Hili 8, the animal economy was made of nearly 90% of domestic fauna throughout its occupation, and cattle contributed around 60% of these remains, while small ruminants such sheep and goats provided the rest [28]. Domestic animal husbandry appears to have primarily contributed to meat consumption, although gazelle bones have been found in all phases, suggesting hunting was also practiced [28]. Little evidence of fish bones or other marine animal remains was found, and this is true for several inland sites [28: 470], with some exceptions [51].

From coastal sites, the faunal assemblages are richer and better studied. Populations on the coast exploited a range of fauna obtained from shallow waters, lagoons, and mangrove forests, including the collecting of shellfish and fishing and hunting sea mammals [52–54]. For example, at Suwayh 3, one of the study sites, marine shells and fishbones were the only types of faunal remains found [55,56]. An architectural structure resembling an earth oven, possibly linked to the smoking/curing of fish, has been reported from Ra’s al-Jinz RJ-2 in Oman during the Late Umm an-Nar period [57]; the possibility of cured fish used for the creation of surplus or as a product of exchange has also been discussed [57–59]. Some coastal sites have evidence of wild hunted animals including gazelle, oryx and dromedary camels [27,28: 474]. Among the domestic animals, sheep and goat were the most common, but at others, cattle were an important component of diet [28]. The evidence suggests the use of mixed subsistence practices at coastal sites, particularly during the Umm an-Nar period [30]. Isotopic analysis of human teeth from EBA sites have *δ*^13^C values indicative of a mixed C_3_-C_4_/marine diet, with an exception of a single site, Umm an-Nar Island, off the coast of the UAE, which shows a more marine-based diet [60]. Thus, despite increased sedentism and the growth of oasis agriculture by *c*. 2500 BC, subsistence strategies in southeastern Arabia varied between settlements depending on their access to different resources. The use of lipid residue analysis can elucidate the presence of terrestrial versus marine products in vessels, providing further insight into potential differences in vessel use at coastal versus inland sites.

### 1.3 Early Bronze Age exchange networks: organic products in pottery

In addition to imported pottery from Mesopotamia and the Indo-Iranian borderlands that was in circulation in southeastern Arabia from the early third millennium BC, exchange networks in the Persian Gulf region grew from c. 2600 BC onwards, and pottery vessels from Bahrain and the Indus region are also found at settlements and tombs [17,61–64]. Softstone vessels locally produced in southeastern Arabia, but also imported from southeastern Iran, were also exchanged inter-regionally during this period [65,66]. Texts from the Sargonic and Ur III periods (c. 2300–2000 BC) mention commodities entering Mesopotamia from toponyms referred to as ‘Dilmun’ (eastern Saudi Arabia/Bahrain), ‘Magan’ (southeastern Arabia and southeastern Iran), and ‘Meluhha’ (the Indus region), including copper, tin, stone, semi-precious stones, as well as organic products [67–69]. Sargonic (c. 2350–2200 BC) texts make limited mention of Mesopotamian goods going into Magan, but there are references to barley, wool, textiles/garments, perfumes, oils and hides, exchanged for copper and diorite stone [69,70: 37–39]. During the Ur III period (c. 2100–2000 BC), when according to Mesopotamian sources, Magan became a major player in Gulf trade, the merchandise moving from Babylonia to Magan consisted of barley/beer/bread, finished textiles, wool, and perfumed oil, exchanged for copper, diorite, and luxury products (the latter likely originating from Iran and the Indus region), such as carnelian, lapis lazuli, softstone vessels, gold, tin, ivory, mangrove wood, *sissoo* wood, as well as exotic animals [70: 58–59; 71]. A single tablet from Ur also refers to the import of 20 litres of an unidentified plant, and 10 litres of the “garlic of Magan” from Magan [70: 58].

There is uncertainty as to whether the imported pottery (or softstone vessels) found in southeastern Arabia were imported for their perceived value or for their contents (or both) [72,73]. It has been suggested that vessels from Mesopotamia might have contained the same substance across several chronological periods, owing to their specific morphological attributes and relatively small volumes (0.5-1 litre), specifically, perfumed plant oils [70: 114–117]. The movement of sesame oil from Mesopotamia is specifically referenced in texts [70: 95, 73,74: 17], and ‘aromatic lard’ is also mentioned in a Sargonic tablet in reference to an expedition to Meluhha [70: 83]. Specific details of the organic products being moved from Iran are not known, but imported pottery such as black-painted Emir ware [75] and incised grey wares [76], are found in Umm an-Nar mortuary contexts; and local imitations of such pottery are also found [77]. Indus artefacts are found in southeastern Arabia from *c*. 2600 BC, particularly during *c.* 2400–2100 BC [17,40,78], and continue appearing into the early second millennium BC [40: 77, 79–81]. One of the most extensively studied Indus-related artefacts found at sites in the Oman peninsula are Black-Slipped Jars (Fig 2), which are large transport vessels, likely linked to the movement of a specific commodity from the Indus region [82–84]. Proposed commodities include dairy products such as clarified butter [71], pickled vegetables, fruit, honey, wine, indigo, or even staples such as grain [85,86]: 97, but such suggestions are based primarily on ethnographic comparisons have not yet been supported by any chemical analysis. The presence of other utilitarian Indus pottery, ornaments, seals, weights and figurines, and Indus-style objects produced with locally available clay at sites in southeastern Arabia [32,86–89] has been linked to the presence of Indus populations residing in Oman, possibly to access copper resources [70,89].

Archaeological evidence of other imported organic products at third millennium BC sites in southeastern Arabia include wood from the Indo-Iranian region [90]; bitumen from northern Mesopotamia [91,92]; ivory from Central or South Asia [61,80,93] and linen textiles, either locally-produced or imported [94].

## 2. Study sites and materials

The main focus of the study was to investigate the contents and uses of mid-third millennium BC Umm an-Nar pottery and imported Indus Black-Slipped Jars from different inland and coastal sites in southeastern Arabia using lipid residue analysis. We also were able to study imported Mesopotamian pottery and locally-produced pottery vessels from early levels at Hili 8 (late fourth-early third millennium BC; Hafit and early Umm an-Nar period).

We analysed 179 potsherds from eight sites (7 settlements, 1 tomb). The sites selected include Bat (particularly the Settlement Slope contexts) [43,95–97], Dahwa 7 [98], Hili 8 [17,40], Hili North Tomb A [77], Kalba 4 [64], Mukhtru [99], Salūt-ST1 [100] and Suwayh 3/Khor Bani Bu Ali SWY-3 [55,56]. The archaeological sites selected occupy different chronological periods within the EBA and beyond, and the sample assemblage included a single fragment dating to the Wadi Suq period from Hili 8. These sites were situated in varied environments, however most are located inland along wadi valleys or on the hillsides of the al-Hajar mountains, while Kalba 4 and Suwayh 3 are the only coastal sites in the study (Table 1; Fig 1). A description of each site and the details of potsherds selected for analysis are provided in S1 File. In several cases, the potsherds that have been studied originate from older excavations conducted in the region (for example, at Hili 8, Hili North Tomb A and Suwayh 3) and the pottery from some of these have been previously subject to detailed ceramic analysis [17], as has the pottery from Salūt-ST1 [32]. Some potsherds from Suwayh 3 (n = 12), Mukhtru (n = 6) and a single sherd from Kalba 4 were from surface contexts. These samples were analysed but have not been included in the final interpretations due to the absence of contextual information. While the sample sizes for Dahwa 7, Hili North Tomb A and Kalba 4 are low, these sites are discussed in combination with all sites to cover overall trends. Necessary permits were obtained for the study, which complied with relevant regulations.

**Table 1:** Study sites and number of potsherds with associated chronological periods (when known) analysed in this study.

Site name	Site code	Period							
		Hafit: 3200-c. 2800 BC	Early Umm an-Nar: c. 2800−2500 BC	Middle Umm an-Nar: c. 2500−2200 BC	Middle-Late Umm an-Nar: c. 2500−2000 BC	Late Umm an-Nar: c. 2200−2000 BC	Umm an-Nar (precise chronological classification not known): c. 2800−2000 BC	Wadi Suq: c. 2000−1600 BC	Total
Hili 8	HIA	8	1	3		13		1	26
Hili North Tomb A	HNA					8			8
Bat	BAT			10	2	3	4		19
Salūt-ST1	SLT			69					69
Mukhtru	MKT				21				21
Dahwa 7	DH7			8					8
Kalba 4	K4						11		11
Suwayh 3	SW3					5	12		17
**Total**		8	1	90	23	29	27	1	**179**

The studied assemblage included imported, regional and locally-produced vessels, as well as three fragments with an indeterminate fabric (Table 2). The imported vessels studied were from Mesopotamia (n = 8; from Hili 8) and the Indus region (n = 32; from different sites), as well as a single Fine Grey Iranian vessel from Makran (from Hili North Tomb A). Regional pottery (n = 27) consisted of pottery designated as Fine Red Omani ware (FR-OM), also labelled as céramique fine rouge “omanaise”[17], Umm an-Nar Fine Red Ware [32,98] or Fine Red Ware [64] in different publications. Found in tombs as funerary pottery [43] as well as settlements [17,32,64] across many sites across the Oman peninsula, Fine Red Omani Ware is homogeneous in its petrographic and chemical composition across multiple examples studied, but its production area and workshop locations are not yet known [17: 109–110]. The distinctive features of this production type include argillaceous or fine silty beige or pale orange fabric, deep tool marks on the internal surface with fine smoothing of the external surface, red-orange slip and black-brown pigment. Forms produced from Fine Red Omani Ware include suspension vessels, short-necked bi-conical vases and necked pots [43,77]. Locally-produced pottery (n = 107) consists of Sandy Wares such as Sandy Red ware, Beige Sandy ware [17,32], Domestic Ware [97], Sandy buff ware and Sandy grey/red ware [98], all of which are hypothesised to be produced and used at the settlement level for domestic purposes. Sandy wares include a wide range of fine sandy fabrics that are beige yellow, white-greenish, dark orange, red or brown, with smoothing on the outer surface and occasional brown wash or brown-painted decoration [17]. The mineralogical and petrographic compositions of Sandy Wares are characterised by elements from sedimentary carbonaceous environments with varying quantities of ophiolitic rock fragments, at least in part produced in the Hili region [17: 39; 101]. Typical forms of this ware include globular open-mouth jars and pots, large jars and suspension vessels [17,32,43,98].

**Table 2:** Numbers of fragments of imported, regional and local pottery studied per site.

Site name	Origin/Type	Imported/Regional/Local	Number of fragments
Hili 8	Mesopotamian	Imported	6
	Indus	Imported	4
	Fine Red Omani ware	Regional	4
	Sandy Red Hili ware	Local	12
Hili North Tomb A	Mesopotamian	Imported	1
	Makran	Imported	1
	Fine Red Omani ware	Regional	4
	Sandy Grey Hili ware	Local	2
Bat	Indus	Imported	2
	Fine Red Omani ware	Regional	1
	Domestic ware	Local	16
Salūt-ST1	Indus	Imported	18
	Fine Red Omani ware	Regional	14
	ST1-Sandy ware	Local	36
	Indeterminate		2
Mukhtru	Sandy ware	Local	21
Dahwa 7	Indus	Imported	1
	Sandy grey/buff ware	Local	7
Kalba 4	Indus	Imported	7
	Fine Red ware	Regional	3
	Indeterminate		1
Suwayh 3	Mesopotamian	Imported	1
	Fine Red Omani ware	Regional	1
	Sandy red or grey ware		15

Different types of fragments, such as rims or the top body fragments (n = 25); body fragments (n = 122) and bases or bottom body fragments (n = 25) were selected for analysis in this study. Of the Sandy wares, vessel forms represented mostly included jars, pots and globular jars (n = 68), likely used for domestic activities, but crucially, no presence of charring or sooting clouds indicating vessel heating over a fire were identified on any of the vessel fragments, which would support their use as cooking pots. Of the Fine Red Omani Ware from settlement contexts, such as at Hili 8, Bat, Salūt-ST1, Dahwa 7 and Suwayh 3, vessels forms represented included jars, pots, and globular jars (n = 27). From Hili North Tomb A, the only funerary assemblage studied, all the Fine Red Omani wares were necked pots (n = 4). The Mesopotamian pottery studied included pots (n = 6) from Hili 8 (Hafit/Early Umm an-Nar) and one bottle from Hili North Tomb A (Late Umm an-Nar). Indus vessels comprised entirely of Black-Slipped Jars (n = 32), all dating to the Umm an-Nar period except one fragment from Hili 8 dating to Period III, the Wadi Suq period. Out of the studied assemblage, 24% (n = 46) had indeterminate vessel forms. For details, see S1 File.

## 3. Methods

### 3.1 Sample retrieval, lipid extraction and analyses

The external surfaces of potsherds (n = 179) were removed with a modelling drill to exclude any exogenous lipids, as per established protocols [102]. Drill bits were cleaned via ultrasonication in dichloromethane (x 3), and were changed between each use. For each sample, *c.* 2–3 g of the potsherd was ground to a fine powder in a solvent-cleaned mortar and pestle. Extractions were conducted immediately after obtaining the pottery powder to minimize lipid loss [see 103]. As most of the pottery studied was from older excavations, it was not possible to study sediment samples originally surrounding the pottery as controls.

Lipids from all the potsherds (n = 179) were extracted via methanolic sulphuric acid extraction alongside process blanks according to established protocols [104,105], but with minor modifications. Prior to the extraction, 10 μL of an internal standard, *n*-triacontane (*n*-C_30_, 1 mg·mL^-1^ in *n*-hexane) was added; this internal standard was used instead of *n*-tetratriacontane (*n*-C_34_) due to the co-elution of minor contaminants (silanes) present in the cyclohexane used. Then, 4 mL of methanol (HPLC grade) was added to the powdered samples, sonicated for 15 min and acidified with concentrated sulphuric acid (800 μl). The samples were heated for 4 hours at 70 °C, and after cooling, lipids were extracted with cyclohexane (3 × 2 mL), centrifuged (3000 rpm, 5 min), and finally concentrated under a flow of nitrogen. All the lipid extracts were analysed directly by gas chromatography-mass spectrometry (GC-MS) to quantify and identify the different organic compounds in the extracts. The instrumental parameters for GC-MS analysis are described in detail in S2 File.

A selection of fragments with relatively higher lipid concentrations or unusual lipid profiles (n = 38) from Hili 8, Hili North Tomb A, Salūt-ST1 and Bat were also analysed via solvent extraction [106] to test for the preservation of triacylglycerols and wax esters. Remaining available potsherd fragments (1–2 g) were cleaned with a modelling drill and ground to a powder in a solvent-cleaned mortar and pestle. Then, 20 μL of an internal standard *n*-tetratriacontane (*n*-C_34_, 1 mg·mL^-1^ in *n*-hexane) was added, and 10 mL of a mixture of dichloromethane/methanol (2:1 v/v; HPLC grade) was added to the powder and sonicated. After centrifugation, the supernatant was evaporated to dryness and dissolved in 500 μL of DCM/MeOH to obtain the total lipid extract (TLE). Aliquots of the TLE were trimethylsilylated (N,O-bis(trimethylsilyl)trifluoroacetamide, 50 μL, 70°C, 60 min) and submitted to analysis by GC-FID and GC-MS, detailed in S2 File.

### 3.2 Compound-specific isotopic analyses

Compound-specific isotopic analysis was conducted on a selection of the acidified methanol extracts. Fifty-one samples from specific vessel types (e.g. BSJs) and with relatively higher concentrations of fatty acids were selected from Hili 8 (n = 11), Salūt-ST1 (n = 19), Bat (Settlement Slope, n = 9), Mukhtru (n = 5) and Kalba 4 (n = 7). Stable carbon isotope values of methyl palmitate (C_16:0_) and methyl stearate (C_18:0_), derived from precursor fatty acids, were measured by GC-C-IRMS following existing procedures [107]; details are provided in S2 File. GC-C-IRMS measurements were carried out at Institute of Environmental Science and Technology of Autonomous University of Barcelona. Differences in fatty acid biosynthesis between ruminant adipose and mammary tissues, as well as of those of non-ruminants create differences in the stable carbon isotope values of fatty acids [108,109]. Ruminant adipose, ruminant dairy, non-ruminant and aquatic fats are distinguished using absolute ranges compared to modern references, as well as by using differences in the carbon isotope values between C_16:0_ and C_18:0_ fatty acids (Δ^13^C = δ^13^C_18:0_ - δ^13^C_16:0_). Results were plotted against δ^13^C values of modern reference terrestrial fats from India [110], Switzerland [111], Kazakhstan [112], Jordan [113], Japan [114], Libya and Kenya [115], as well as C_3_ plant oils [116], published elsewhere. Fatty acid δ^13^C values of modern references fats from the study region are not yet available.

Figures, data summaries were generated, and statistical analyses (S3 File) were performed using Rstudio version 4.0.3. Kruskal-Wallis tests with post-hoc Wilcoxon rank sum tests were used since the data were non-parametric.

## 4. Results

### 4.1 Lipid preservation

**Fig 3:**
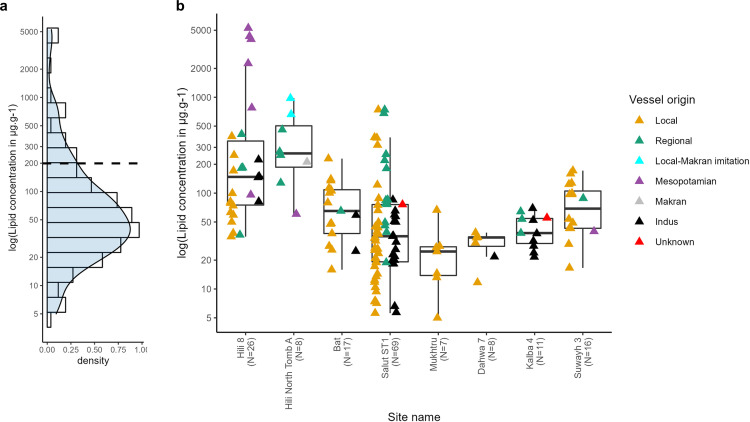
a) Histogram and density plot of lipid concentrations (on log scale in μg g^-1^) from all study sites. Dashed line indicates mean value. b) Lipid concentrations (on log scale in μg g^-1^) of locally-produced Sandy wares, regional Fine Red Omani Wares, Mesopotamian, Indus, Iranian Painted-Grey Ware from Makran, local Makran-imitation, and pottery with unknown origin across all chronological periods categorised by study sites. Lipid concentrations below the interpretable range (5 μg g^-1^) were excluded from the plot.

The archaeological sites chosen in this study cover a range of different environments and terrains including coastal locations to sites within wadis or alluvial plains and/or foothills of the al-Hajar mountains. Lipid preservation in pottery is variable between sites, possibly influenced by environmental factors, taphonomic processes or vessel usage. For example, potsherds from Hili 8 and Hili North Tomb A had relatively high lipid concentrations compared to all other sites, while potsherds from Mukhtru exhibited poor lipid yields with only 7/21 potsherds (29%) yielding lipid concentrations above the interpretable range (above 5 μg g^-1^) (Table 3). The average lipid concentration for all sites was 200 μg g^-1^ but the median value was 49.8 μg g^-1^ (Table 3; Fig 3a). Of the solvent-extracted sherds from Hili 8, Hili North Tomb A, Bat and Salūt-ST1 (n = 38), only a small proportion of extracts (12/38, 31.6%) had total lipid extracts (TLEs) above interpretable thresholds, i.e., 5 μg g^-1^, from Hili 8 and Bat (S4 File).

**Table 3:** Number of potsherds analysed, % lipid recovery (i.e., % of vessels with lipid concentrations above 5 μg g^-1^) and mean lipid concentrations (in μg g^-1^) of pottery from different sites included in the study.

Site name	Site location	Site type	Number of potsherds analysed		% Lipid recovery	Mean lipid concentration (μg/g)
Hili 8	Wadi	Settlement	26		100	752.0
Hili North Tomb A	Wadi	Funerary	8		100	377.2
Bat	Wadi	Settlement	19		90	80.2
Salūt-ST1	Low terrace adjacent to wadi	Settlement	69		100	89
Mukhtru	Wadi	Settlement	21		29	8.9
Dahwa 7	North Batinah plain	Settlement	8		100	30.2
Kalba 4	Coast	Settlement	11		100	43.3
Suwayh 3	Coast	Settlement	17		94	75.6
**TOTAL**			179		90.5	200

Differences in lipid concentrations were also observed between vessels with different origins (Fig 3b). Analysed Indus Black-Slipped jars had smaller ranges of, and lower mean and median lipid concentrations compared to other vessel types such as locally-produced Sandy Wares and regional Fine Red Omani vessels. A Kruskal-Wallis test supports differences in lipid concentrations among vessels with different origins (χ^*2*^*(5)* = 38.9, p = < 0.001); with pairwise comparisons using Wilcoxon rank sum test supporting differences in lipid concentrations between locally-produced Sandy Wares and Fine Red Omani pottery (p = < 0.001); Fine Red Omani sherds and Indus Black-Slipped Jars (p = < 0.001); Mesopotamian pottery and Sandy Wares (p = 0.001); and Mesopotamian pottery and Indus Black-Slipped Jars (p = < 0.001). Such differences are possibly linked to degree of vessel porosity and vessel usage.

### 4.2 Molecular characterisation

**Fig 4:**
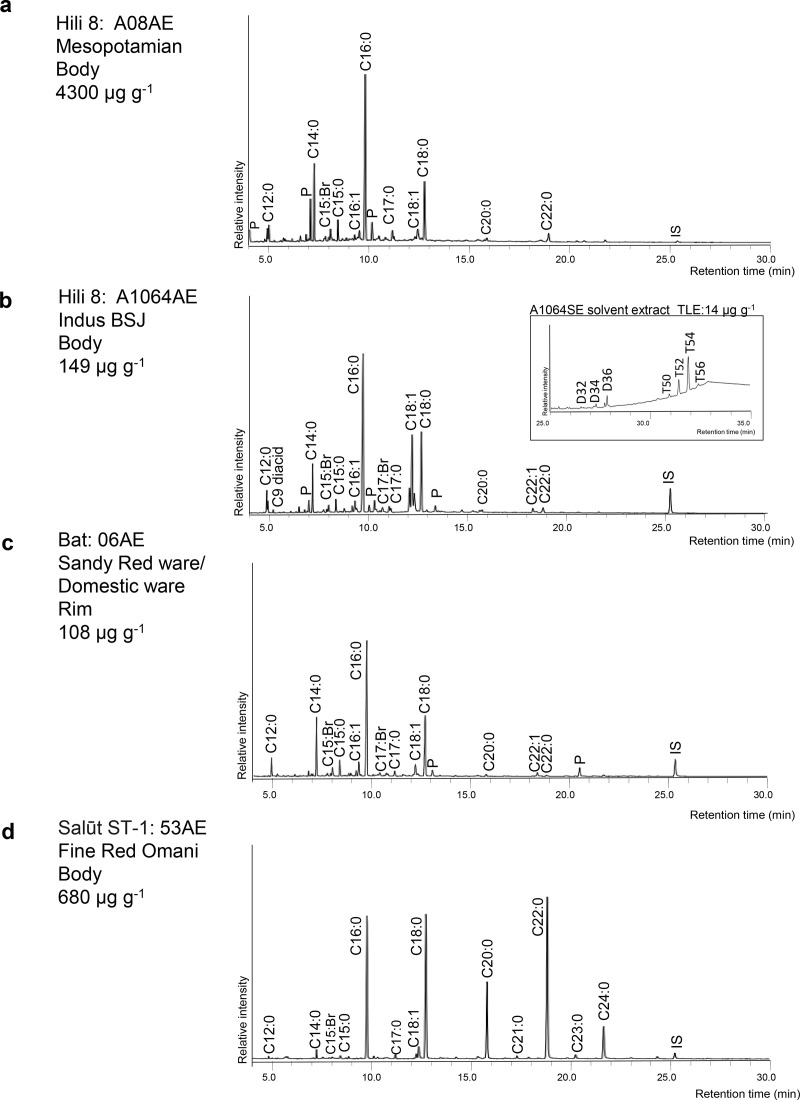
Examples of lipid profiles of pottery acidified methanolic extracts investigated in this study, including imported vessels such as a) Mesopotamian pottery and b) Indus Black-Slipped Jars from Hili 8; locally-produced pottery such as c) domestic ware/sandy red ware from Bat, and d) regional Fine Red Omani vessel from Salūt-ST1. a) and c) are indicative of degraded animal fats; with c) containing relatively high abundances of docosenoic acid (C22:1) over docasanoic acid (C22:0). b) suggests the presence of plant oil as indicated by the presence of unsaturated triacylglycerols in its solvent extract (see inset), and d) is indicative of a plant product. Cn:x indicates fatty acid with n carbon atoms and x double bonds; IS: Internal Standard; P: phthalate, Br: branched-chain fatty acid; diacid: α,ω-dicarboxylic acids, D: diacylglycerol, T: triacylglycerol.

Excluding extracts from surface contexts (twelve fragments from Suwayh 3, two fragments from Mukhtru, and a single fragment from Kalba 4, n = 15) and those with lipid concentrations below 5 μg g^-1^, a total of 147 extracts were considered for further molecular characterisation. A suite of different lipid classes was detected, the most abundant of which were *n*-alkanoic acids, ranging from C_12:0_ to C_18:0,_ dominated by tetradecanoic acid (C_14:0_), hexadecanoic acid (C_16:0_), and octadecanoic acid (C_18:0_). Most extracts (120/147, 81.6%) also contained odd-chain fatty acids such as C_15:0_ and C_17:0_, as well as branched-chain fatty acids such as C_14_, C_15_, and C_17_. Peaks of unsaturated fatty acids such as hexadecenoic acid (C_16:1_), octadecanoic acid (C_18:1_), docosenoic (C_22:1_)(126/147; 85.7%) and in some instances, eicosenoic acid (C_20:1_)(6/147, 4.1%) were also detected in vessels, as were α,ω-dicarboxylic acids (63/147, 42.8%). Other lipid classes detected include aliphatic lipids such as *n*-alkanes in very low abundances in 15 extracts (15/147, 10%), of which four had distributions indicative of petroleum-derived *n*-alkanes [117]. Of the remaining, only one extract had a clear odd-over-even profile, while six had odd-chain *n*-alkanes only, and four had no clear distributions. Ketones or other heating markers were not detected in any of the extracts. Phthalates were found in varying amounts in the samples, in relatively higher concentrations in extracts from Hili 8, Hili North Tomb A and Suwayh 3. Such compounds are relatively common in archaeological pottery and are related to vessel contact with plastic bags [117]. Other contaminants, such as homosalate, a component in sunscreen, were detected in two extracts. Although such contaminants are identifiable, their presence can mask other relevant compounds of archaeological interest. Fig 4 provides chromatograms for representative lipid profiles within the studied assemblage.

Of the solvent extracts with interpretable lipid concentrations, *n*-alkanoic acids (C_10:0_-C_18:0_), dominated by hexadecanoic and octadecanoic acid were detected in all 12 extracts. Cholesterol derivatives and trace quantities of monoacylglycerols were detected in 9 extracts; trace amounts of diacylglycerols and triacylglycerols (TAGs) were also found in 5 extracts (HIA02, HIA07, HIA08, HIA14, HIA15). Two extracts from Hili 8 (HIA802 and HIA1064) demonstrated very high survival of unsaturated TAGs (over 50% of the Total Lipid Extract) (Fig 4B). In these two extracts, T_54_ (triolein) dominated, and the T_52_ and T_50_ peaks comprised octadecanoic and hexadecanoic acid moieties which were identified based on their specific mass spectra and retention time [previously reported in 22].

### 4.3 Molecular interpretation

The majority of the lipid profiles (120/147, 81.6%) are indicative of degraded terrestrial animal fats as indicated by the presence of mid-chain fatty acids, odd- and branched-chain fatty acids, and low abundances of long-chain fatty acids ranging from C_20:0_ to C_28:0_ [118]. Additionally, α,ω-dicarboxylic acids are present in several extracts (63/147, 42.8%), mainly C_9_ (azelaic acid), although a few samples (n = 10) contained a broader range from C_6_ - C_15,_ indicative of the degradation of unsaturated fatty acids [119]. As dicarboxylic acids occur in both animal, plant and aquatic products, and cannot be confidently traced to their precursor unsaturated fatty acid [120–122], such compounds are not particularly diagnostic. Furthermore, the presence of docosenoic acid (C_22:1_) in nearly half the vessels (in some instances 21/145; 14.2%, more than docosanoic acid, C_22:0_) may indicate evidence of seed oils from the *Brassicaceae* family [123,124], or also marine organisms [125]. However, others have suggested this compound can be present as a laboratory contaminant [e.g., 124], so the presence of this compound is treated with caution.

Secure evidence for plant products is found in five extracts (5/147 = 3.4%), namely, two Indus BSJs (HIA802, HIA1064) from Hili 8, a necked FR-OM pot from Hili North Tomb A (HNA410) and two FR-OM vessels (SLT53 and SLT54) from Salūt-ST1. The Indus BSJs from Hili 8 (HIA802 and HIA1064) had unsaturated fatty acids, octadecadienoic acid (C_18:2_), unsaturated TAGs and stigmasterol in the solvent extracts, as well as octadecadienoic acid (C_18:2_) and higher concentrations of octadecenoic acid (C_18:1_) over octadecanoic acid (C_18:0_) in the acid extracts. Both extracts, but particularly the presence of the unsaturated TAGs and stigmasterol in the solvent extracts, suggests the presence of plant products such as plant oils in these vessels [126]. Similarly, the FR-OM vessel from Hili North Tomb A had higher concentrations of octadecenoic acid (C_18:1_) over octadecanoic acid (C_18:0_), hexadecenoic acid (C_16:1_), octadecadienoic acid (C_18:2_), hydroxy acids and a P/S ratio of 5.3, collectively providing evidence for the presence of a plant product [121]. Two FR-OM vessels from Salūt-ST1 (SLT53 and SLT54) had high abundances of long-chain fatty acids (LCFAs) relative to other fatty acids, particularly docasanoic acid (C_22:0_) (Fig 4D). These did not occur with their fatty alcohol counterparts, but odd-chain LCFAs were present, which have previously been reported in West African archaeological Nok pottery [127]. High concentrations of docasanoic acid have been reported in cowpea (Vigna unguiculata) [127,128], but the presence of this legume is not attested in the archaeobotanical record in southeastern Arabia, and the extracts from Salūt-ST1 also had high concentrations of hexadecanoic and octadecanoic acid. As LCFAs originate in leaf or stem epicuticular waxes and are also reported on the surface of plant leaves, sheaths, stems and fruits, seed oils, seed coats, flowers, bark and husks, in this context, they can only be interpreted as a general indicator for plant products [129–132].

Other extracts have unusual profiles, but less secure evidence for plant products. Four extracts from Salūt-ST1, specifically three Indus BSJ fragments (SLT01, SLT74 and SLT79) and a Sandy Red Ware pot (SLT22), had high abundances of tetradecanoic acid (C_14:0_) relative to other fatty acids [23], but lacked other plant biomarkers. High abundances of dodecanoic (C_12:0_) and tetradecanoic acid (C_14:0_) are reported in palm kernel oils [133], which is considered an important resource in region [12], but dodecanoic acid (C_12:0_) was not found in these extracts. The absence of dodecanoic acid (C_12:0_) also makes it challenging to calculate fatty acid ratios which have previously been used to support the presence of seed oils or a plant source [134,135]. While it is possible that dodecanoic acid (C_12:0_) was preferentially leached from the vessel compared to longer-chained compounds, the high abundances of octadecanoic acid (C_18:0_) in the extracts may also suggest the mixing of other components in these vessels, making a clear interpretation challenging.

Additionally, a small number of extracts (n = 7) had low concentrations of a series of *n*-alkanes which might be linked to the presence of plant waxes [129–132]. Of these, four were locally-produced Sandy Red Hili vessels (HIA123AE, HIA126AE, HIA127AE and HIA129AE) from Hili 8, which had odd-chain *n*-alkanes maximizing at C_25_ (HIA123AE), C_27_ (HIA127AE and HIA129AE) or C_29_ (HIA126AE), with average *n*-alkane chain-lengths (ACL) ranging from 27.1 to 27.7 [136]. Distributions of *n*-alkanes maximizing at C_25_ are unusual, characteristic of submerged and floating aquatic plants [132,137], and in the case of HIA123AE, its P_aq_ value [137] is 0.33, consistent with emergent macrophytes or plants that grow in shallow water [137]. However, given that these profiles from Hili 8 were lacking typical odd-over-even distribution of *n*-alkanes, our interpretations are made with caution. From Salūt-ST1, SLT47, a locally-produced Red Sandy Jar had an odd-over-even *n*-alkane distribution maximizing at C_29_, with ACL of 27.7 and Carbon Preference Index (CPI) [136] of 1.3. Other vessels from Salūt-ST1 had odd-chain *n*-alkanes (SLT50 and SLT73), with ACLs of 21. As petroleum-derived *n-*alkanes have CPIs close to 1 [117], this suggests that we cannot be confident that the *n*-alkanes in extracts from Salūt-ST1 originated from plants.

Finally, four extracts, all belonging to Fine Red Omani vessels (HIA148 from Hili 8, HNA377 from Hili North Tomb A, and K4.27 from Kalba 4), had relatively high abundances of octadecenoic acid (C_18:1_) above or equal to octadecanoic acid (C_18:0_), along with the small peaks of octadecadienoic acid (C_18:2_), tentatively linking them to plant products [138]. Unfortunately, the extracts were lacking oxidation products or other secure plant biomarkers. As it has been noted that octadecenoic acid is present in not only plant oils, but also animal fats, is susceptible to oxidisation and thermal degradation and may be a sign of contamination [117], these profiles, although unique, are not identified as plant products with certainty.

### 4.4 Compound-specific isotopic analysis

**Fig 5:**
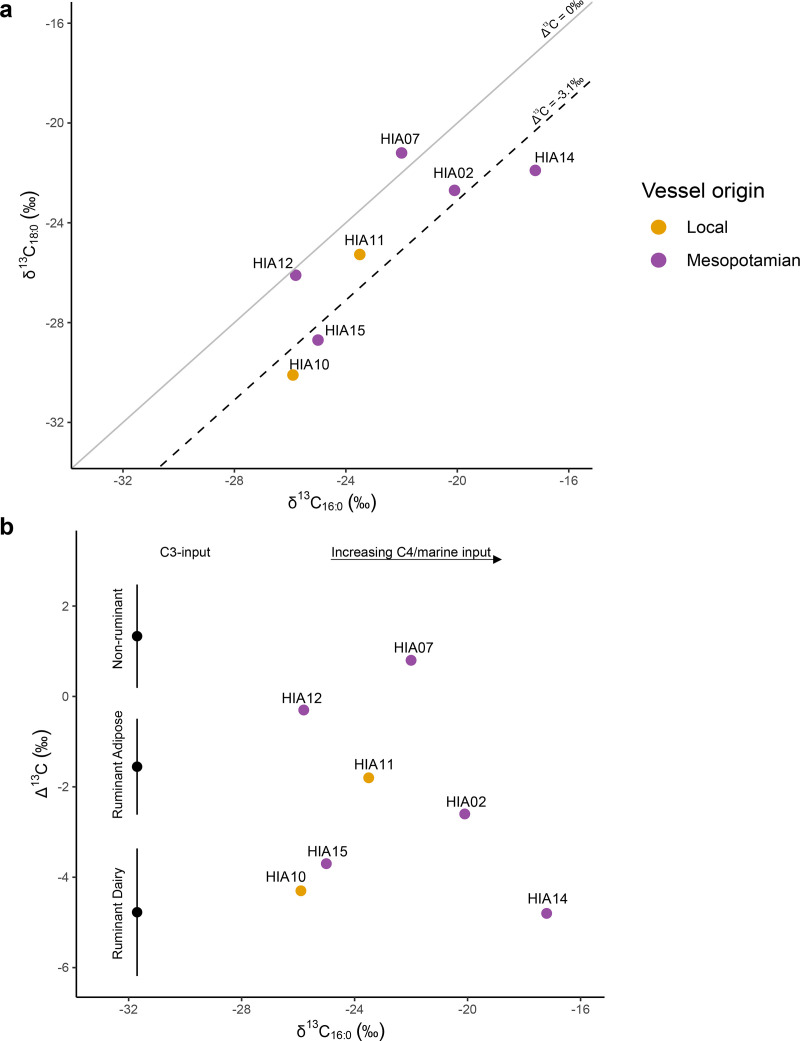
Scatter plots showing δ^13^C values of a) C_18:0_ and C_16:0_ fatty acids and b) Δ^13^C (C_18:0_ - C_16:0_) and δ^13^C values C_16:0_, measured from Mesopotamian and local Sandy Red Hili ware vessels from Hili 8 dating to the Hafit and Early Umm an-Nar periods (Period I). In b), ranges depicted represent the mean ± 1 s.d. of Δ^13^C values of previously published modern reference terrestrial fats from India [110]; Switzerland [111], Kazakhstan [112]; Jordan [113]; Japan [114], Libya and Kenya [115].

**Fig 6:**
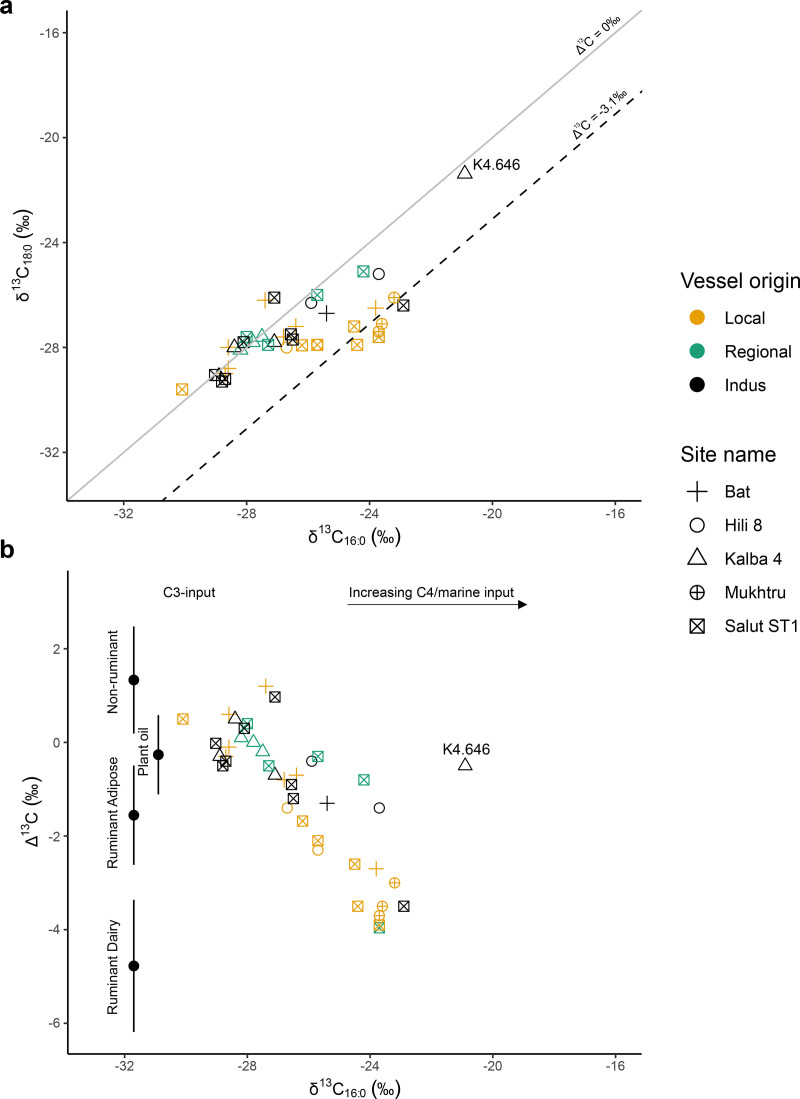
Scatter plots showing δ^13^C values of a) C_18:0_ and C_16:0_ fatty acids and b) Δ^13^C (C_18:0_ - C_16:0_) and δ^13^C values C_16:0_, measured from Middle and Late Umm an-Nar vessels from Hili 8, Bat, Salūt-ST1, Mukhtru and Kalba 4. In b), ranges depicted represent the mean ± 1 s.d. of Δ^13^C values of previously published modern reference terrestrial fats from India [110]; Switzerland [111], Kazakhstan [112]; Jordan [113]; Japan [114], Libya and Kenya [115]; as well as C_3_ plant oils such as olive, sesame, walnut, argan and moringa oil [116].

Stable carbon isotopic values (δ^13^C) of the major fatty acids (C_16:0_ and C_18:0_) from 48 extracts from Hili 8, Salūt-ST1, Bat, Kalba 4 and Mukhtru range from −17.2‰ to −30.1‰ for C_16:0_ and −21.2‰ to −30.1‰ for C_18:0_. The samples included 8 samples dating to the Hafit or early Umm an-Nar period (5 Mesopotamian pots, 3 local Sandy Red Hili Ware globular jars) from Hili 8 (Fig 5), while the rest date to the Middle or Late Umm an-Nar periods (Fig 6). The fatty acid isotopic values exhibit an isotopic shift due to environmental factors such as aridity, warm climate and varying abundances of C_3_/C_4_ plants in the animals’ diets [e.g., 115,139] and are thus classified using their Δ^13^C (δ^13^C_18:0_ - δ^13^C_16:0_) values. Additions from marine products can also be detected through δ^13^C values, although mixing with terrestrial fats can mask these signals [140].

The obtained Δ ^13^C values range from −4.8‰ to 1.2‰, with 9 samples (19%) falling within reference ranges for dairy products and 18 samples (37.5%) with values consistent with ruminant carcass fats, which include the meat of sheep/goat, cattle, and wild ruminants such as dromedary camels or deer. Twelve samples (25%) have values that fall in-between ranges for ruminant carcass fats and non-ruminant fats, while others (7/48, 14.6%) have values consistent with non-ruminant fats. Two extracts (HIA07, a Mesopotamian vessel from Hili 8 and K4.646, an Indus Black-Slipped Jar from Kalba 4) have δ^13^C values that fall within the range of marine products [104,140]. As indicated by Fig 6b, several extracts have Δ ^13^C values that are also consistent with C_3_ plant oils [116]; a topic further explored in Section 3.2. In the future, a more detailed assessment of the molecular presence of aquatic products will be carried out to assess their presence in the samples.

The δ^13^C_16:0_ values range between 17.2‰ to −30.1‰; the enrichment of values suggesting the input of C_4_ plants or marine input in the diet of ruminants and/or non-ruminants. Lake sediment sequences from Ra’s al-Khaimah, UAE indicate that by the sixth millennium BC, the area was dominated by mixed C_3_-C_4_ vegetation, with an increase in C_4_ grassland from 2500 BC, peaking at 2100 BC with an intense phase of aridity [141,142]. Domesticated C_4_ crops are absent during the EBA in the region, and the presence of sorghum (*Sorghum bicolor),* although reportedly found at Hili 8 [46], has been called into question on morphological grounds [e.g., 12: 235, 48, 143]. However, a range of C_4_ plants such as xeromorphic shrub and grasslands, halophytic sand grasses, and sedges, are found in the region in different environmental zones, including inland deserts, coastal saline flats and wadis, often along with C_3_ shrubs and vegetation [144]. These plants serve as important sources for pasture and grazing in the region even today [145].

From the Hafit and early Umm an-Nar periods, isotopic values from lipid extracts from Hili 8 indicate that locally-produced Sandy Red Ware vessels contained ruminant adipose fats and dairy products. Vessels originating from Mesopotamia have varying δ^13^C values, indicating the presence of ruminant adipose fats (HIA02), or ruminant dairy products (HIA14), and their δ^13^C_16:0_ values suggest that the ruminants they originated from had an increased C_4_ plant or marine input in their diet during their lifetime [109,115]. One vessel has fatty acid-specific isotopic values that are consistent with a marine signal (HIA07).

## 5. Discussion

The combined lipid and compound-specific isotopic results from different vessels originating in Early Bronze Age sites across southeastern Arabia presented here provide direct evidence of organic products used in ceramic vessels in the region. The results provide insight into the contents of domestic pottery as it was adopted during the Umm an-Nar period, highlighting the importance of re-situating pottery foremost as containers with use-life [146] involved as part of everyday alimentary practices [147], and beyond. The detection of ruminant meat, dairy, non-ruminant fats, plant products and their mixtures in vessels demonstrates the use of natural resources in ceramics, as well as vessel-usage and culinary practice. These are topics so far little-explored in Arabian archaeology. The results also raise questions about whether organic products within specific imported vessels can be detected, and provide the first promising results about the movement of organic products in vessels as part of exchange networks, specifically Mesopotamian vessels from Hili 8 during the Hafit and early Umm an-Nar period, and Indus BSJs found across several coastal and inland southeastern Arabian sites from the mid-third millennium BC onwards.

### 5.1 Pottery linked to the use of pastoral products in the Early Bronze Age southeastern Arabia

The adoption of new technologies or innovations are linked to specific social needs that they may fulfil [148], their ‘fittingess’, i.e., how new technologies work within broader interconnections between people and things [149], and pre-existing technologies that contribute to their development [4,148]. Pottery technology in southeastern Arabia was adopted at a relatively fast pace during the early-mid third millennium BC, within the context of other pyrotechnologies, such as metalworking, as well as the movement of imported pottery and possibly craftspeople from the Indo-Iranian region and Mesopotamia [16–20,150]. Other types of container or basket technologies likely existed as part of alimentary practices in prehistoric southeastern Arabia, such as the production of containers or baskets using date palm fronds [151] or animal skins and intestines, but archaeological evidence for them has not survived. Based on the results of this study, it may be postulated that the successful adoption of domestic pottery was linked to its ‘fittingness’ with already established container technology used in an incipient agrarian society, as well as a long-established Arabian lifeway of pastoralism, which encompassed a broad range of animal husbandry practices that potentially included mobility and some amount of agriculture [e.g., 152,153].

The molecular evidence suggests that the source of most of the extracts was animal fats, and isotopic results indicate the presence of ruminant adipose fats, for example, from cattle, goat/sheep, or wild ruminants such as dromedary camels, gazelle and ibex (18/48, 35%) and dairy products (9/48; 22%). Available zooarchaeological evidence, particularly from inland sites, is consistent with these results. Domestic cattle, sheep and goat played a vital role in the EBA subsistence economy [28]. At Hili 8, cattle constituted the about 60% of the domestic animals throughout the Early Bronze Age. During the Hafit period (Period I), a majority of the domestic fauna (80%) were slaughtered before they reached the age of four years, which points to their main use as a source of meat, particularly for cattle and sheep [28: 480]. In contrast, the kill-off pattern for goats suggests that about half of them lived beyond 36 months and nearly 20% lived longer than four years, suggesting their use for secondary products such as dairying and wool production [28: 480]. Although we cannot be certain of the species from which the milk originates, the isotopic evidence from this study confirms the presence of dairy products (e.g., milk, cheese, butter, yoghurt and/or clarified butter) in both Mesopotamian and local Sandy Red Hili vessels during the Hafit or Early Umm an-Nar period from the site of Hili 8 (Period I and IIa-b). This evidence suggests the presence of dairying in southeastern Arabia at least by c. 3000 BC.

Other evidence of dairy products in vessels exists from both Salūt-ST1 and Mukhtru during the Middle Umm an-Nar period, but it is not yet attested at Bat (Settlement Slope contexts). At Mukhtru the samples with evidence for dairy products constitute the majority of analysed samples, and at Salūt-ST1 they comprise a smaller percentage, although they are detected in different vessel types, in locally-produced Sandy Red Omani pots, a Fine Red Omani jar, and an Indus BSJ (Fig 6b). Similarly, ruminant carcass fats are also found in a range of different vessel forms, although the use of wild ruminants for meat cannot be excluded. Evidence of a dairy and pastoral economy is consistent with the faunal remains at Salūt-ST1 [23]. Unfortunately, due to the poor preservation of faunal remains at Bat and Mukhtru, it is not possible to compare the results with zooarchaeological remains at the sites. Stable carbon isotopic analyses of faunal teeth from other Umm an-Nar sites in the UAE suggest that browsers such as sheep/goat had a predominantly C_3_ plant diet, while grazers such as cattle had a mixed C_3_-C_4_ plant diet, with some wild ruminants in coastal regions indicating a high C_4_ diet [60]. The *δ*^13^C_16:0_ values obtained from lipid extracts suggest a mixed input of C_3_ and C_4_ plants in the diet of ruminant animals, agreeing with previous enamel isotopic results. It is notable that extracts falling within dairy reference ranges during the Middle Umm an-Nar period present more positive *δ*^13^C_16:0_ values. Such results are similar to those obtained in the Indus region [154], but not in other arid regions of the world [e.g., 113,155], perhaps pointing at shared dairying foodways amongst connected groups.

**Fig 7:**
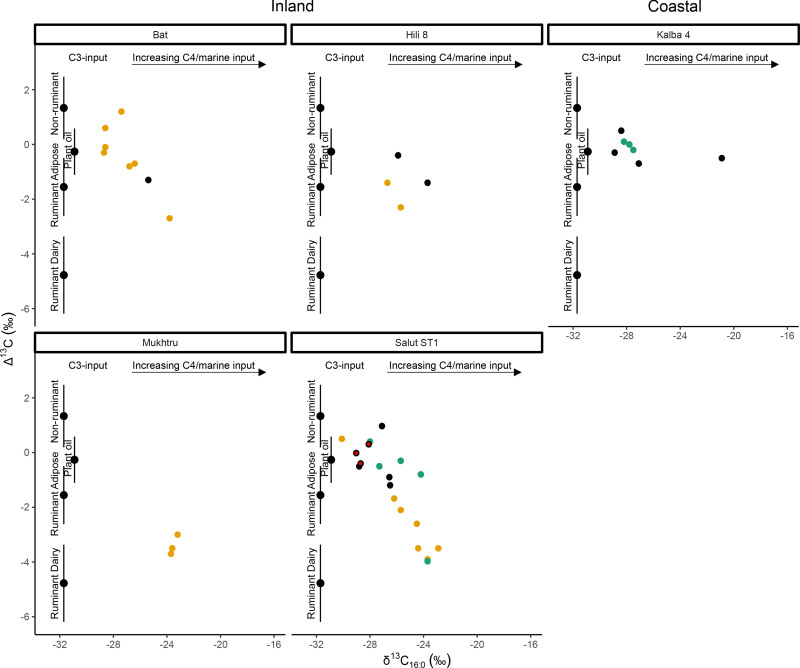
Δ^13^C (C_18:0_ - C_16:0_) and δ^13^C_16:0_ values, measured from Middle and Late Umm an-Nar local (filled orange circles), regional (filled green circles) and Indus Black-Slipped Jars (filled black circles) from Hili 8, Bat, Salūt-ST1, Mukhtru (inland) and Kalba 4 (coastal). Three Indus Black-Slipped Jar fragments from Salūt-ST1 belong to the same vessel [23] and are marked by red circles with a black outline. Ranges depicted represent the mean ± 1 s.d. of Δ^13^C values of previously published modern reference terrestrial fats from India [110]; Switzerland [111], Kazakhstan [112]; Jordan [113]; Japan [114], Libya and Kenya [115]; as well as C_3_ plant oils such as olive, sesame, walnut, argan and moringa oil [116].

Comparisons between vessel use at inland and coastal sites are challenging due to low sample sizes and minimal isotopic data from coastal sites (Fig 7). From Suwayh 3, only 5 of 17 vessels (4 local Sandy Red Omani vessels and one Mesopotamian vessel) were considered for detailed interpretation; these vessels had lipid profiles typical of degraded terrestrial animal fats, but their fatty acid isotopic values were not obtained. In contrast, no faunal evidence of terrestrial ruminant animals has been reported at Suwayh 3 [56], which may suggest that pottery might have had specific uses at the site, however a detailed examination of the presence of aquatic products and fatty acid isotopic values for extracts from Suwayh 3 are required to better distinguish the terrestrial versus marine source of the fats. Similarly, from Kalba 4, of the 10 vessel fragments analysed, only three were Fine Red Ware vessels, and the rest were Indus Black-Slipped Jar fragments (Fig 7). While lipid profiles from the Fine Red Ware vessels suggest the presence of degraded animal fats, their fatty acid *δ*^13^C values fall in-between ranges of ruminant adipose fats and non-ruminant fats and within those of plant oils, evading clear interpretation (see Section 5.2).

The results suggest a correlation between animal fats, meat and milk products and the use of pottery, particularly at inland southeastern Arabian EBA sites. As a highly concentrated source of energy, animal fat is an important resource in arid contexts, particularly in times of food stress [151,156], and animal milk and blood are vital in pastoralist communities in other parts of the world [157]. In Oman, it has been observed that traditional food practices dictate that meat is only consumed on special occasions, however, preserves of animal fat and protein are prepared for use for longer periods of time [158]. Thus, while it is possible that lipid residues may not reflect the day-to-day consumption or relative importance of different foodstuffs [e.g., 159], they provide a vital starting point to think about connections between pastoral lifeways and pottery. It is possible that animal fats, meat, and dairy could have been stored over longer periods of time in EBA pottery, which has important implications for how we think about EBA subsistence, pastoralism, and how domestic pottery was conceived of and used.

### 5.2 Plant products in vessels?

Lipid extracts provide evidence of the use of plant products in a small proportion of vessels (3.4%) from the studied assemblage, with fourteen other extracts (9.5%) with unusual profiles, but not unequivocal evidence for plants. The most secure evidence for plant oils was detected in two Black-Slipped Jar vessels from Hili 8 [22], a Fine Red Omani necked vessel from Hili North Tomb A, and other plant products in two Fine Red Omani vessels from Salūt-ST1 [23]. Extracts with odd-chain *n*-alkanes typical of plant waxes were reported from a small number of Sandy Red Hili vessels from Hili 8, and unusual lipid profiles were reported from Indus Black-Slipped Jar fragments from Salūt-ST1, as well as Fine Red Omani vessels from Salūt-ST1, Hili 8 and Kalba 4. A link between the presence of plant products and Fine Red Omani vessels (n = 7) may be suggested; however a larger sample and more secure plant biomarkers are required to test this hypothesis.

Despite the minimal molecular evidence for plant products, a large proportion of the fatty acid *δ*^13^C values from the analysed vessels (at least 20/51, 39%) fall within ranges observed for modern C_3_ plant sources and overlap with modern references for ruminant adipose and non-ruminant fats [116] (Fig 6b). It is possible they represent the mixing of different resources (plant and/or animal)[e.g., 160], and it is difficult to attribute a single source to these extracts. For example, both the Fine Red Omani vessels from Salūt-ST1 with strong molecular evidence for plant products (SLT53 and SLT54) have *δ*^13^C values that fall within reference ranges of ruminant adipose fats and ruminant adipose/non-ruminant fats, respectively (S4 File).

The ephemeral evidence of plants in the lipid extracts is likely caused by a number of factors, including the fact that plant products contain considerably less lipids than animal products; as well as the fact that plant and aquatic products with high concentrations of unsaturated fatty acids quickly degrade to resemble profiles of terrestrial animal fats; creating an inflated picture of the animal-based component of vessel contents [161,162]. The minimal presence of non-ruminants in the faunal record from inland sites in southeast Arabia [28] and absence of modern reference data adds further complexity to the interpretation. However, the use of equids or other non-ruminants as part of dietary practices cannot be ruled out [23].

Agricultural products such as cereals, legumes and fruits, grown in date-palm gardens have been attested at Early Bronze Age settlements in southeastern Arabia [46–50]. The presence of grinding stones in settlement contexts also suggests the processing of plants for consumption [e.g., 100]. Unfortunately, the detection of cereal-derived compounds via lipid residue analysis is challenging due to the exceptional conditions required for their survival [163], thus it is not possible to be confident of their presence or absence in the studied vessels. This likely leads to an underrepresentation of potential cereals being stored and processed in ceramic containers. It is plausible that the gathering of fruits, seeds, shoots, leaves and tubers from numerous wild species along with cereal consumption would have been a part of food practices, and the lipid results likely provide insight into the use of plants that rarely survive in the macrobotanical record in southeastern Arabia.

### 5.3 Products in Mesopotamian vessels during the Hafit and Early Umm an-Nar periods

The site of Hili 8 provides some of the earliest evidence of imported and locally-produced pottery in southeastern Arabia, with a small assemblage of Mesopotamian vessels and locally-produced black-on-red pottery found during Period I, i.e., the Hafit and Early Umm an Nar period (c. 3200−2800 BC) [40]. The imported Mesopotamian vessels analysed in this study indicate relatively high lipid concentrations and the presence of varied animal fats, including dairy products, ruminant adipose fats, and marine products. The range of δ^13^C_16:0_ values indicate that animals from which these fats were derived subsisted on forage composed of C_3_ and C_4_ plants (A10, A11), or mainly of C_4_ plants or marine vegetation such as seaweed (e.g., A02 and A14). In contrast, locally-produced vessels from the same period at Hili 8 have evidence of dairy products and ruminant carcass fats with evidence of mixed C_3_ and C_4_ plant input (Fig 5).

High lipid yields in the small sample of imported Mesopotamian pottery may be linked to sustained use of high fat-yielding products [164], and the diversity of resources detected in them is intriguing, possibly indicating the arrival of different products from afar. Notably, however, none of these vessels have indications of plant oils, which presumably were one of the products coming from Babylonia into Magan according to Mesopotamian textual sources [70: 97]. However, the lipid content of plant-based products is at least tenfold lower than animal-based products, leading to potentially biased results [163]. Similarly, evidence for barley or beer is difficult to detect, given the limited preservation of compounds characteristic of cereals such as alkylresorcinols in archaeological lipid extracts [163]. ‘Aromatic lard’ is another product mentioned as an export in Mesopotamian texts in reference to sea-based expeditions [70: 83], suggesting that some of the animal fats detected in the vessels may indeed have a non-local source. The presence of products with a marine signal in one Mesopotamian pot (A07) is particularly pertinent as it provides the first direct evidence of products arriving from afar in vessels, at least from the coast. However, we cannot exclude that the products detected may not reflect the ‘original’ contents of Mesopotamian vessels: given that Hili 8 is an inland site, it is possible that the vessels may have been re-used for different commodities, depending on the way exchange was organised, and the mechanisms by which the vessels arrived at the settlement.

### 5.4 Varied uses of Indus Black-Slipped Jars

Indus Black-Slipped Jars (BSJs) represent a unique class of vessels found across EBA sites in southeastern Arabia [82–84]. The results of this study provide the first direct evidence of the contents of Indus BSJs at different sites, dating primarily to the Umm an-Nar period, with one vessel from Hili 8 dating to the Wadi Suq period (Hili 8 Period III). Combining both the molecular and isotopic results, Indus BSJs appear to have contained different products, including terrestrial animal fats (specifically dairy products, ruminant carcass fats, non-ruminant fats), marine products and plant oils (Fig 6). From Hili 8, two Indus BSJs (A0802 and A1064) have a high percentage of unsaturated fatty acids and unsaturated triacylglycerols, providing strong evidence for the presence of plant oils [22]. Ongoing analysis of the triacylglycerol distribution may be able to provide insight into the source/s of these oils in the future [e.g., 165].

Other BSJs from Hili 8 and other inland sites such as Bat and Salūt-ST1 indicate the presence of degraded animal fats, with inconclusive evidence of plant input suggested by the high relative abundance of C_14:0_ and/or higher abundances of unsaturated fatty acids (C_18:1_ and C_22:1_). While the isotopic results suggest that the source of the animal fats for some BSJs is ruminant carcass fats, many vessels have *δ*^13^C values that are ambiguous and might reflect the mixtures of non-ruminant products, ruminant adipose fats and/or plant oils. A single BSJ vessel from Salūt-ST1 has evidence for dairy products [22].

Indus BSJs from the coastal site of Kalba 4 reflected molecular evidence of degraded animal fats and isotopic evidence of non-ruminant/plant/ mixtures of products for all except one vessel that had *δ*^13^C values consistent with marine products. Given Kalba 4’s location along the eastern coast of the UAE and its connections with maritime exchange networks [64], it is possible that vessel contents of Indus BSJs at Kalba 4 reflect actual imported product/s. However, given the small sample size, it is premature to pinpoint the source of specific imported products at this time, and the analysis of vessels from more coastal sites is required. It is possible the original contents of these vessels when shipped did not correspond to later re-use in southeast Arabia. Instead, the cumulative evidence from inland and coastal sites suggests that BSJs contained commodities such as plant oils, lard, dairy products and marine products over their use-life, reflecting multi-purpose usage.

## 6. Conclusion

Our results demonstrate the link between pottery and pastoral or hunted products as pottery production was widely adopted across southeastern Arabia during the Early Bronze Age. While the growth of pottery technology is synchronous to increasing sedentism and the adoption of oasis agriculture in the region, this study suggests that its uses were connected to herding and hunting, which were ancient, deeply established lifeways in Arabia. This study highlights the pastoral component of the Umm an-Nar period which is somewhat understated in archaeological treatment of the period. The direct evidence of dairy products in some of the earliest-known locally-produced pottery vessels also suggests the existence of dairying economies at least by the early third millennium BC, continuing into the mid-third millennium BC. The presence of plant products in some vessels is noteworthy as plants are generally underrepresented due to their low lipid content but also because they are more susceptible to degradation. These results highlight that a diversity of commodities were used in pottery vessels and likely consumed; providing new ways to interrogate the relationship between pottery and subsistence practices. Our study also reveals crucial new information about the presence of different products in imported Mesopotamian vessels, including dairy products, ruminant carcass fats as well as marine products. Indus Black-Slipped jars from different sites across southeastern Arabia appear to have been used to store a diversity of commodities such as plant oils, ruminant carcass fats, dairy products and marine products. These may indicate the arrival of different products from afar, or multiple uses of vessels throughout their use-life; but it is notable that marine products are detected in two imported vessels, a hint to a potential trend that should be explored in the future.

Our study takes a unique approach by investigating locally-produced, regionally distributed, and imported pottery from different sites across southeastern Arabia as a first step to characterising potential differences in the contents of different vessel types. Future research is required into understanding details of preservation dynamics and their relationship to environmental conditions in the region. The study of a larger sample of vessels, especially from coastal sites, may help elucidate regional variations in vessel-use and dietary practices. Additionally, the creation of a robust modern reference baseline for the region is critical for better interpretation of the isotopic data. In the future, the combination of biomolecular and technological approaches to the study of pottery may help explain the complex pathways that led to its adoption and spread in southeastern Arabia.

## Supporting information

S1 File Details of study sites. (PDF)

S2 File Details of instrumental analyses. (PDF)

S3 File R code for figures and statistical analysis. (PDF)

S4 File Results of GC-MS and GC-C-IRMS analyses. (CSV)

S5 File Translation of article abstract in Arabic. (CSV)
