# Supplementary material for: Identifying pastoral and plant products in local and imported pottery in Early Bronze Age southeastern Arabia
Source: PLoS One. 2025 Jun 11;20(6):e0324661. doi: 10.1371/journal.pone.0324661 (PMC12157666; doi:10.1371/journal.pone.0324661)
Supplement: S1 File — (PDF) [file pone.0324661.s001.pdf]

Supplementary Information 1: Details of study sites

1. *Hili 8*

Located in the al-Ain oases on the western foothills of the Hajar mountains, Hili is a large complex of sites including both settlements and tombs spread across at least 25 ha, located about 150 km east of Abu Dhabi, in the eastern province of the Abu Dhabi Emirate, UAE. Within the Hili complex, the settlement of Hili 8 provides the longest stratified sequence for the Early Bronze Age (EBA) in south-eastern Arabia to the Middle Bronze Age (Wadi Suq period) and possibly the Late Bronze Age (LBA). Hili 8 was excavated by teams led by Serge Cleuziou (†) from CNRS and University of Paris 1 Panthéon-Sorbonne. The principal architectural remains at Hili 8 consisted of the base of a solid, compartmented mudbrick tower, built at the beginning of the Hafit period (Phase la), with further constructions at the beginning of the Umm an-Nar period (Phase IIa) and at the end of the Umm an-Nar period (Phase IIf), as well as ditches, other mud-brick constructions, and wells [1, 2, 3: fig 147] (Figure 1).

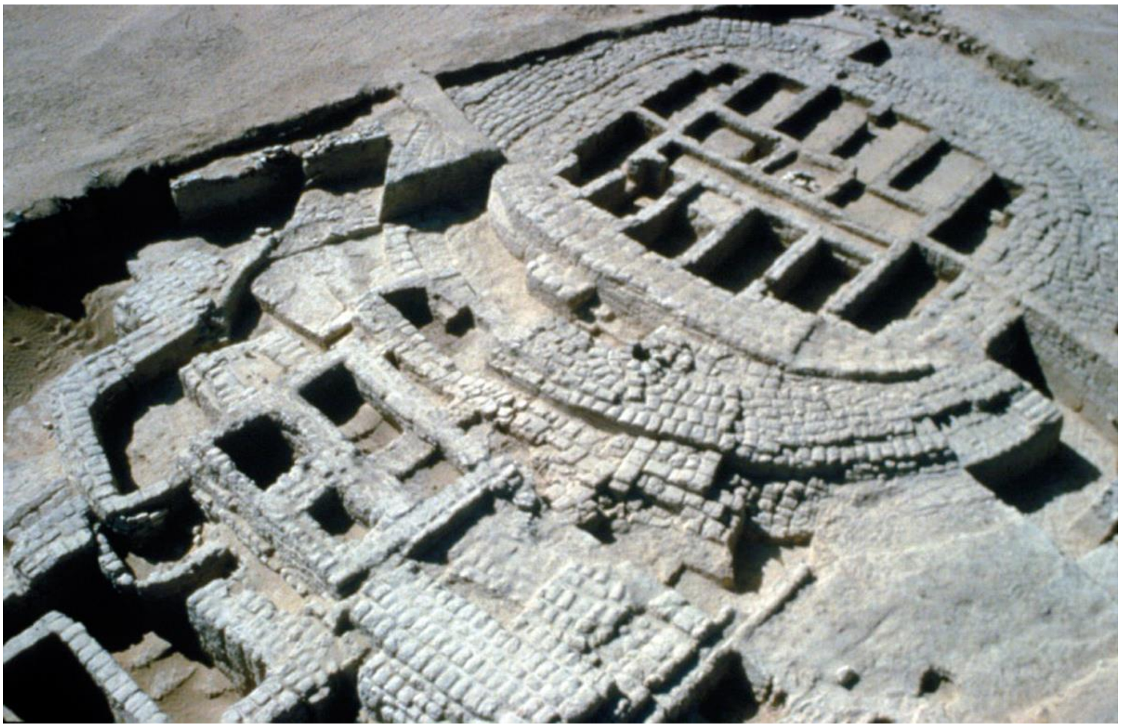

Figure 1: Image of mudbrick constructions at Hili 8. Photo: S. Cleuziou/French Archaeological Mission to the United Arab Emirates.

The samples selected for lipid residue analysis were excavated in the 1970's and 1980's by French Archaeological Mission to the United Arab Emirates. Thirty-two potsherds spanning across Period I (Hafit (n=8), Umm an-Nar (n=17), and Wadi Suq and LBA (n=7) periods were chosen. From the Hafit period, six potsherds were imports from Mesopotamia and two were locally-produced Hili Red Sandy Ware(HI-SR). From the Umm an-Nar period, ten locally-produced Hili Red Sandy Ware (HI-SR), four regionally-produced Fine Red Omani Ware (FR-OM), and three imported Black-Slipped Jars (BSJ) from the Indus Civilisation were analysed. From the Wadi Suq period and LBA periods, three Fine Wadi Suq potsherds, one Indus Black-Slipped Jar fragment and three samples of Coarse Ware were selected for analysis.

1.2. Hili North Tomb A

Tomb A at Hili North is 2 km north-north-west of Hili 8 and consists of a multi-chambered collective burial tomb (Figure 2), excavated by teams led by Serge Cleuzio (†)u with the contribution of Burhardt Vogt. Likely used for no more than 200 years, it is contemporaneous with phases IIf-g at Hili 8 [4, 5]. Tomb A is one of the largest known examples of Umm an-Nar-type monumental circular tombs, which are widespread throughout the Oman peninsula from the second half of the third millennium BC [3, 6, 7, 8]. Built of stones and faced with large ashlars and a single door stone, Tomb A contained four parallel chambers in which more than eighty individuals were buried [5].

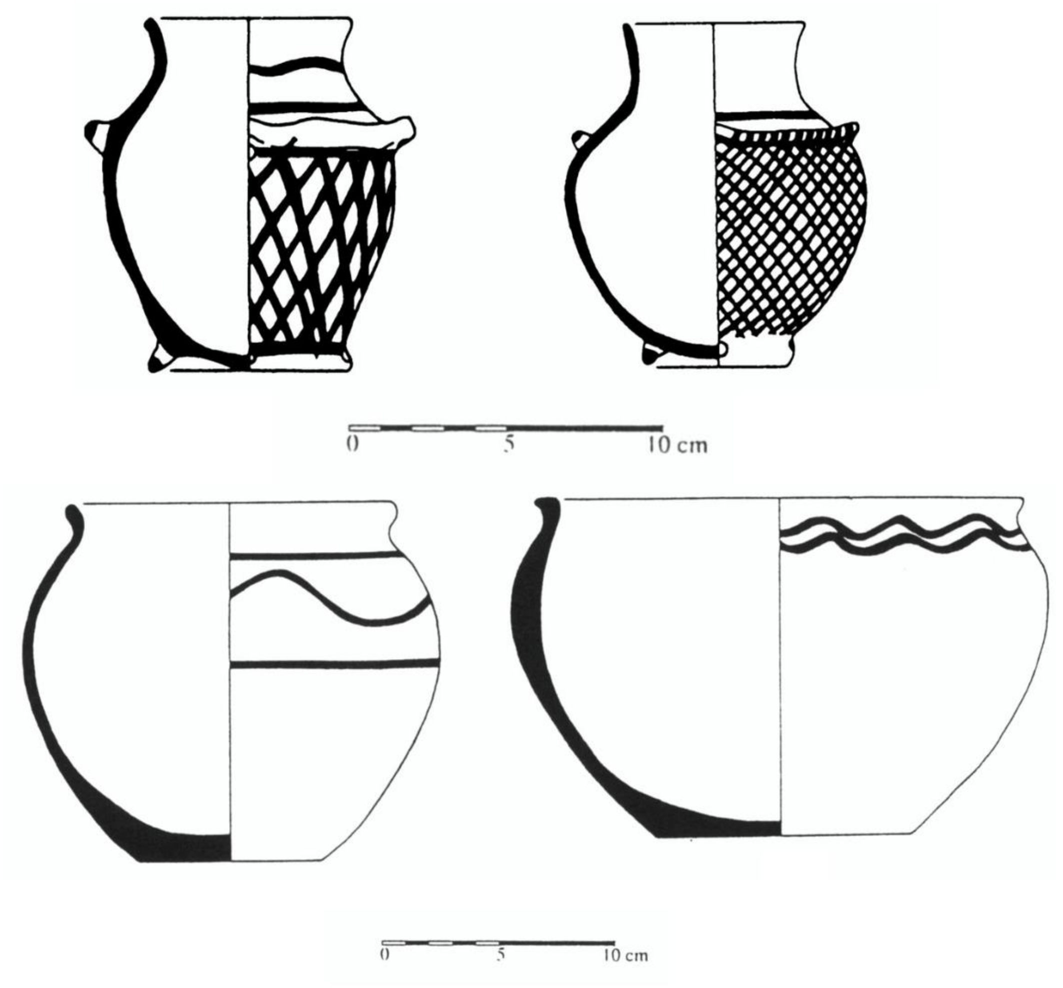

Figure 2: Examples of Hili Sandy Red Ware suspension vessels (top) and jars (bottom) excavated from Hili North Tomb A. Drawings by Hélène David-Cuny.

Nearly 900 pottery vessels were recovered from the tomb, of which about 10% were imported and included a large proportion of Indus and Makran/Sistan pottery (4% and 5% respectively) [9: 172]. However, petrographic and chemical analyses revealed that large fraction of the Makran/Sistan vessels were in fact produced locally [10: 71–72, 11: 85–217]. The rest of the vessels found were also produced locally or regionally, of which 65% were Hili Sandy Red Ware (SR-OM) and 19% were Omani Fine Red Ware (FR-OM) [9: 172].

Eight pottery fragments were chosen for lipid residue analysis from Hili North Tomb A. Of these, two were imported (fragments of a Mesopotamian bottle and a Fine Grey Emir Painted pot) and six were locally-produced vessels (two fragments of Sandy Grey Incised wares produced at or near Hili (SG-IR), and four fragments of Fine Red Omani 'FR-OM' necked pots).

1.3. Salūt ST-1 (SLT)

Salūt ST-1 is a 22 m circular stone tower dating to the Umm an-Nar period (*c*. 2400-2000 BC) near Bisyah, central Oman [12]. The Bronze Age tower excavation was led by the Italian Archaeological Mission to Oman in collaboration with the Office of the Adviser to His Majesty the Sultan for Cultural Affairs. Pottery samples from the excavations at Salūt ST-1 were collected in December 2015.

The tower had a central stone-lined well and was surrounded by a large ditch (11-13m wide and up to 3m deep) with two connecting channels (Figure 3). These features were interpreted as related to water management and storage [12, 13]. In a late phase of the tower's occupation (*c*. 2460–2145 BC), waterborne sediments gradually filled the main ditch, which eventually became used as a dumping area [13]. A wide range of Indus and Indus-related pottery types, including utilitarian pottery and specific forms used for food production, presentation, and storage were recovered from the stratigraphic levels associated with the ditch at Salūt ST-1 [13]. Indus seals and carnelian beads possibly manufactured with non-Indus raw materials were also recovered from another part of the ditch [13, 14]. The presence of a large and diverse assemblage of Indus material culture, as well as local vessels produced with typical Indus shapes has led to the suggestion that Indus potters were living in central Oman, probably by *c*. 2500 BC [14].

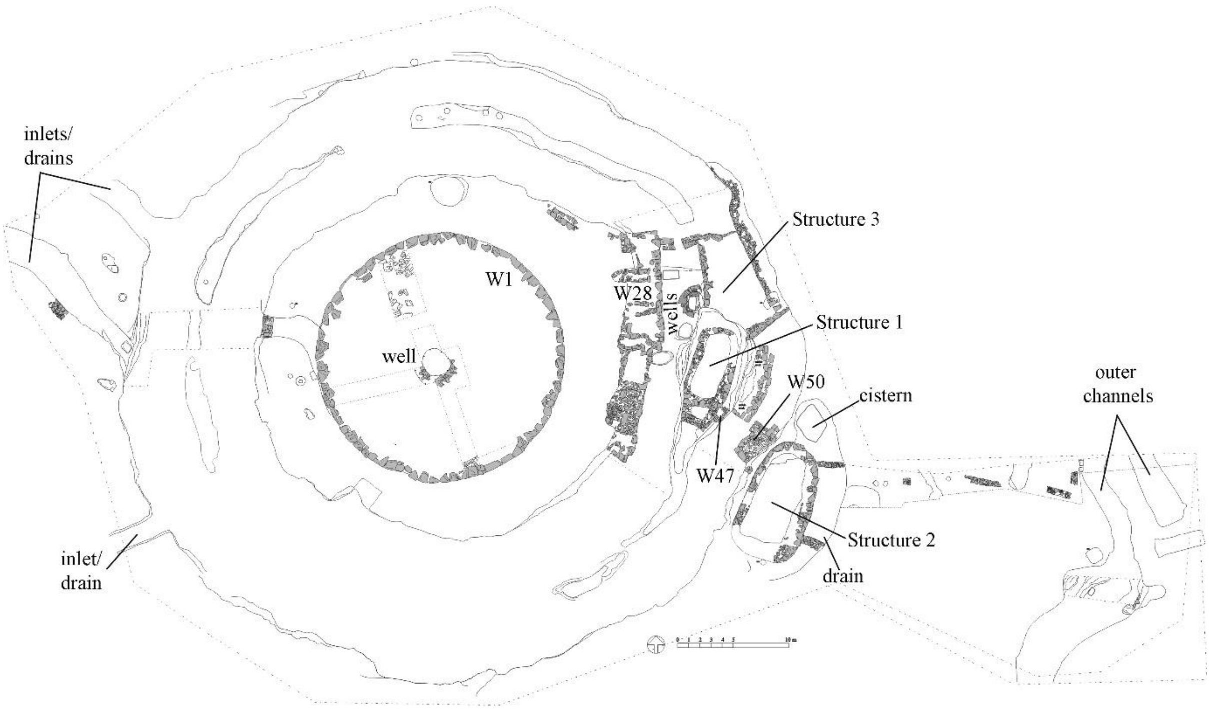

Figure 3: General plan of Salūt ST-1 with features.

Potsherds for lipid residue analysis were chosen from contexts located within the main ditch that contained evidence of ephemeral occupation, demonstrated by hearths in sandy fills and concentrations of pottery. A total of 69 vessel fragments were selected: 14 fragments of Fine Red Omani Ware (FR-OM), 36 fragments of Sandy Red Omani Ware (SR-OM), 18 fragments of Indus Black-Slipped Jars, and one indeterminate vessel (Figure 4).

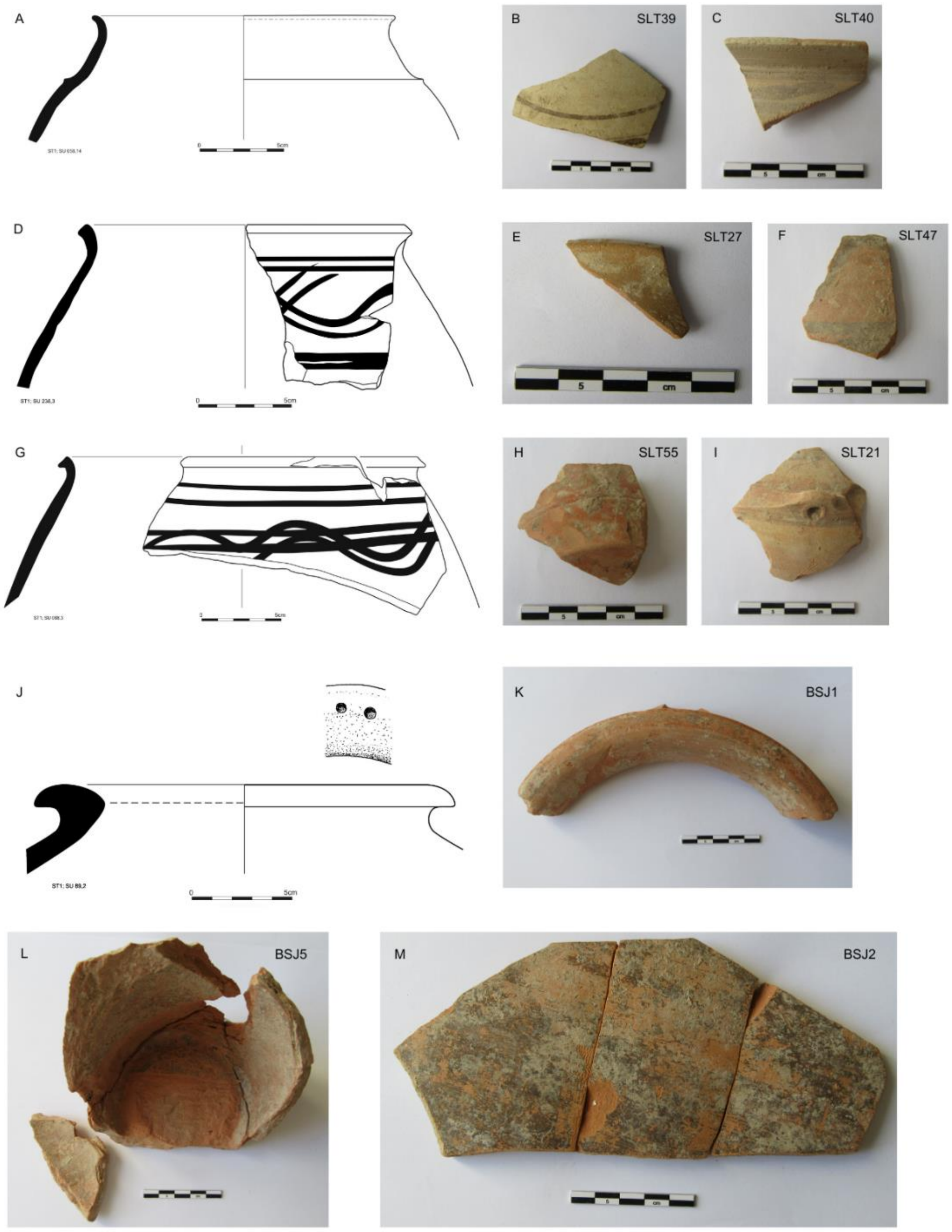

Figure 4: Examples of pottery sampled for analysis from Salūt ST-1, including Sandy Red Omani Wari (A-F), Fine Red Omani Ware fragments (G-I) and Indus Black-Slipped Jars (J-M). Reproduced from [15].

Due to the targeted nature of sampling, state of vessel preservation, and colour of the interior and exterior slip of Indus BSJs at Salūt ST-1, it was possible to determine whether certain fragments of the analysed Indus BSJs belonged to the same vessel. Visual examination of the

Indus BSJs revealed that the analysed assemblage contained at least 5 unique vessels, of which three vessels had multiple fragments [15].

1.4. Mukhtru (MKT)

The pottery fragments obtained from the site of Mukhtru, located in the Al-Mudhaybi region close to Wadi Andam in Central Oman were provided by the Al-Mudhaybi Regional Survey [17]. In an area of approximately 1.2 ha south of the modern village of Mukhtru, several stone walls are visible on the surface (Figure 5). Intensive field-walking surveys revealed a high density of Umm an-Nar pottery at the site, and small-scale excavations revealed structures with stone walls and fireplaces resembling domestic architecture [17] (Figure 5). Radiocarbon dates from charcoal from the fireplaces produced dates between c. 2450-2050 BC. Thus, Mukhtru is one of the few Umm-an Nar settlements found in the Al-Mudhaybi region apart from Al-Khashbah and Al-Qabrayn.

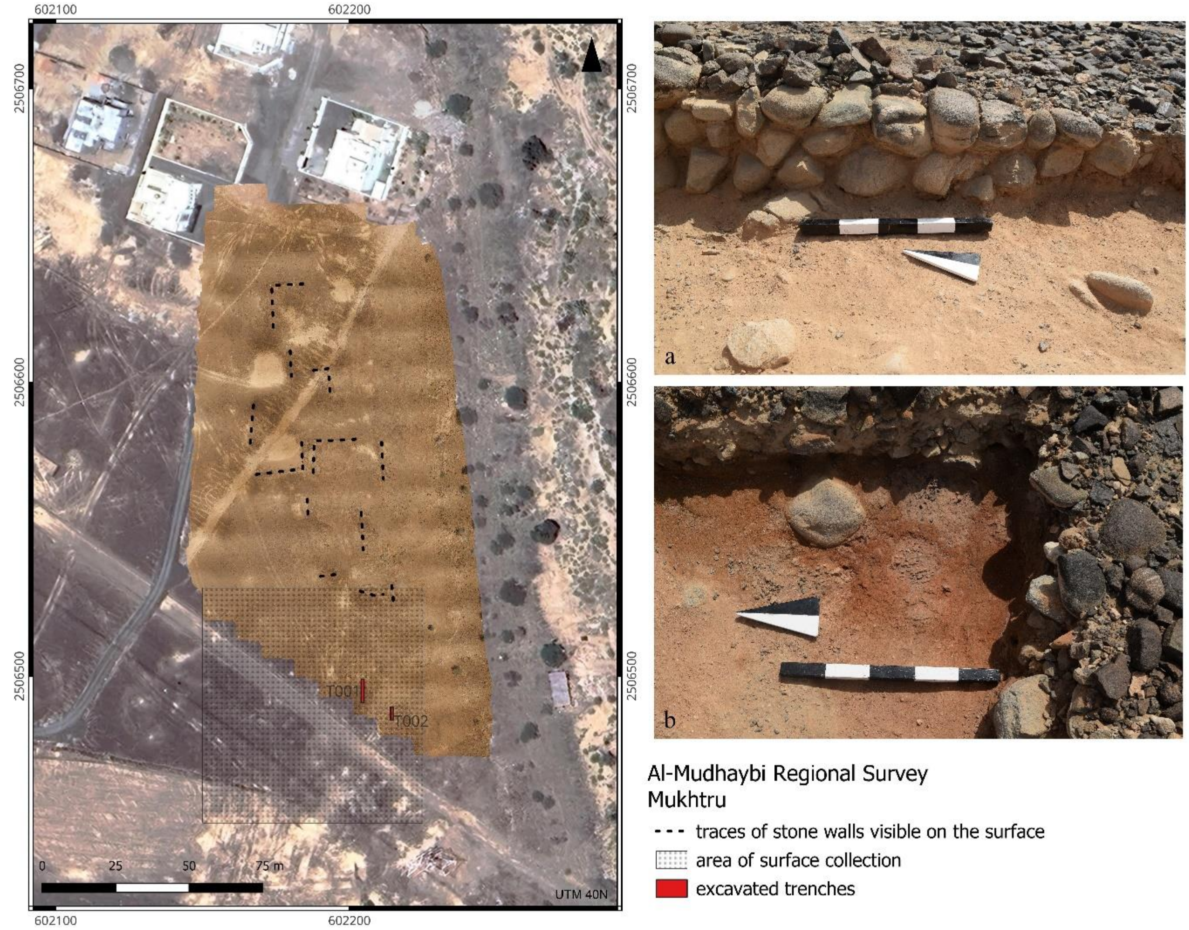

Figure 5: Area with Umm an-Nar period domestic architecture in Mukhtru with excavated stone wall (a) and fire place (b).

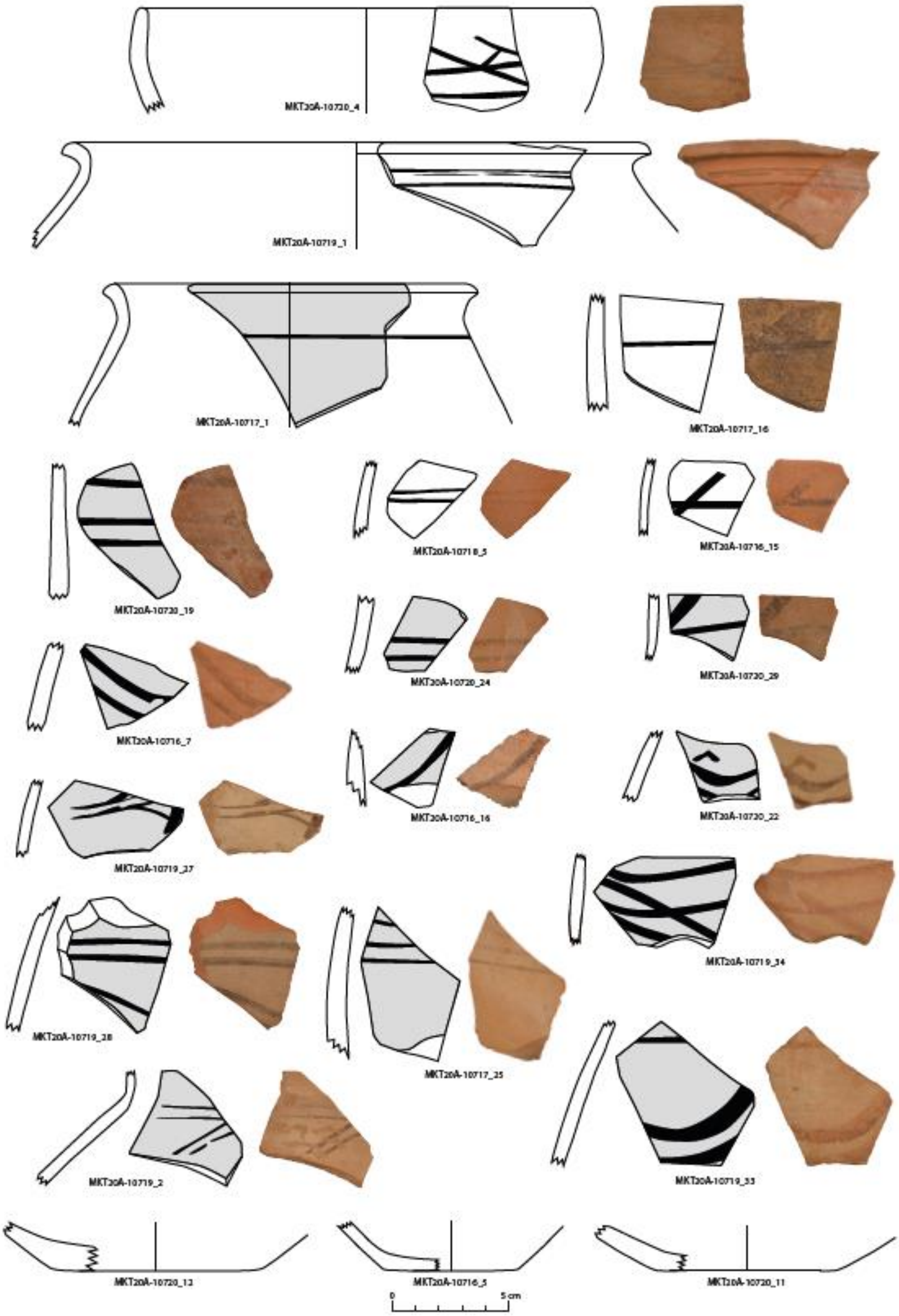

Figure 6: Pottery from Mukhtru sampled for lipid residue analysis.

Twenty-one sherds from the excavations were sampled for lipid residue analysis (Figure 6). These include rim, body and base sherds from small and medium Sandy Ware jars and bowls, produced locally. Many of them are painted in the typical Umm an-Nar style, with straight and wavy lines in black to reddish brown on a red, brown or buff background.

1.5. Bat

Bat is an archaeological landscape located in the Wadi Al Hijr of the Hajar mountains' inner piedmont zone. Archaeological remains are spread across nearly 400 hectares, and date from the Neolithic through to the present day, with substantial remains dating to the Bronze Age [18]. The Bronze Age remains include seven monumental towers, burials and agricultural areas dating as early as the Hafit period (3200-2800 BC), and at least three area of occupational architecture dating to the Umm an Nar period (2800-2000 BC) [18].

Twenty potsherds for lipid residue analysis from Bat were chosen. Samples (n=17)were primarily chosen from the dense architectural remains on the hillside of the Settlement Slope, located in the north of the Bat landscape [18, 19] (Figure 7). The fragments of pottery selected for analysis included a range of Umm an-Nar pottery, some of which could be precisely dated to the Middle (2500-2200 BC) and Late (2200-2000 BC) Umm an-Nar periods [18], and two Indus Black-Slipped Jar fragments. Additionally, one potsherd was selected from al-Khutm [19:62-65], located ca. 3km northwest of Bat; one from Operation A, an extension of the Bat Necropolis, which is located c.500m north [20]; and another from an Iron Age I/II tomb in the Bat landscape. The tomb is located on the al-Ahliyah hill, approximately 400m southeast of the Settlement Slope.

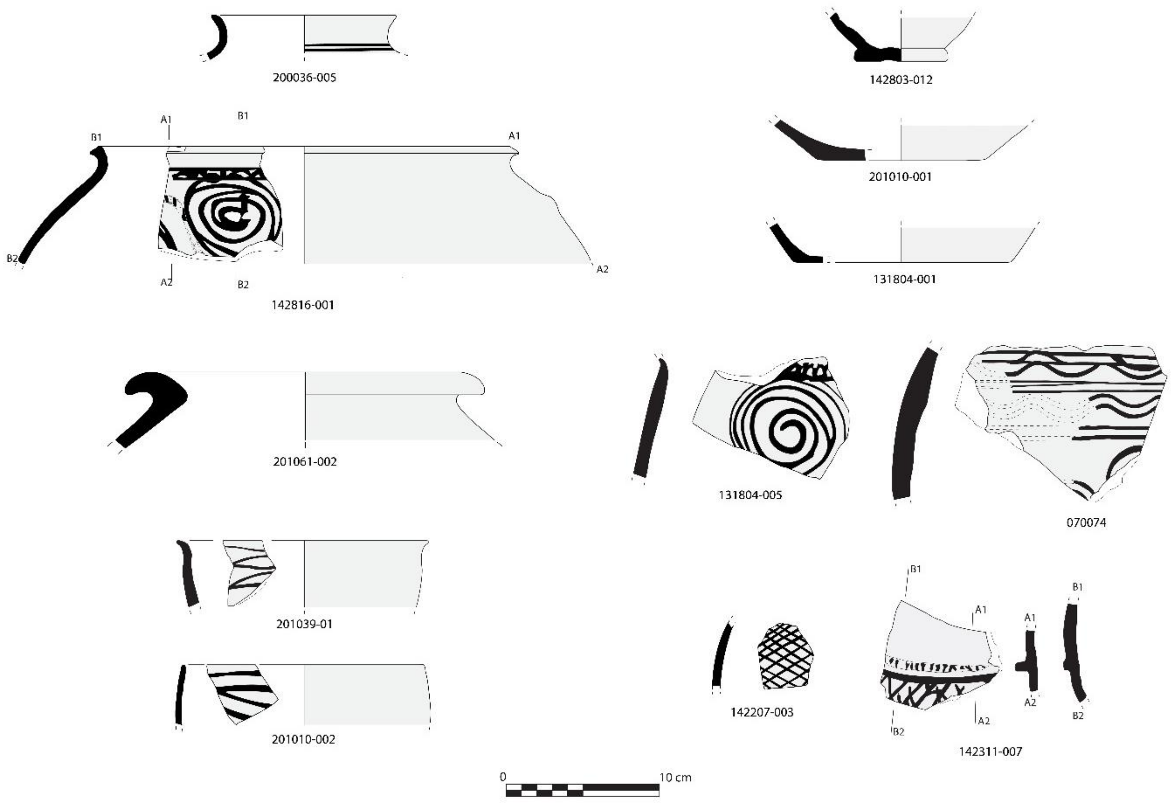

Figure 7: Drawings of some sampled potsherds from Bat. Drawings by Reilly Jensen and Jennifer Swerida.

1.6. Dahwa 7 (DH7)

The Umm an-Nar settlement of Dawha 7 (DH7) is located in the northern al-Batinah region in north-eastern Oman, at the base of the al-Hajar mountains [21]. Excavated by teams led by Khaled Douglas and Nasser Al-Jahwari from the Archaeology Department at Sultan Qaboos University, with the contribution of Kimberly Williams from Temple University, DH7 is about 6.4 ha in size and has at least 31 buildings which date to *c*.2500 B.C [21]. Of the buildings excavated, building S42 was the largest, with six rooms; and an adjoining courtyard and small room that were added at a later phase [21] (Figure 8). The excavators have interpreted the room as a warehouse, as it is separated from other structures in the settlement; contains a large number of potsherds; and it's shape and size do not indicate a domestic function. An equal number of local and Indus wares were found in Building S42, with a

majority of the Indus vessels belonging to Black-Slipped Jars [21]. A majority of Indus BSJ fragments were found in the courtyard (R4), dating to the latest occupation of the building. Radiocarbon dates obtained from building S42 fall within the latter half of the 3rd millennium BC (*c*. 2500-2250 BC)[21].

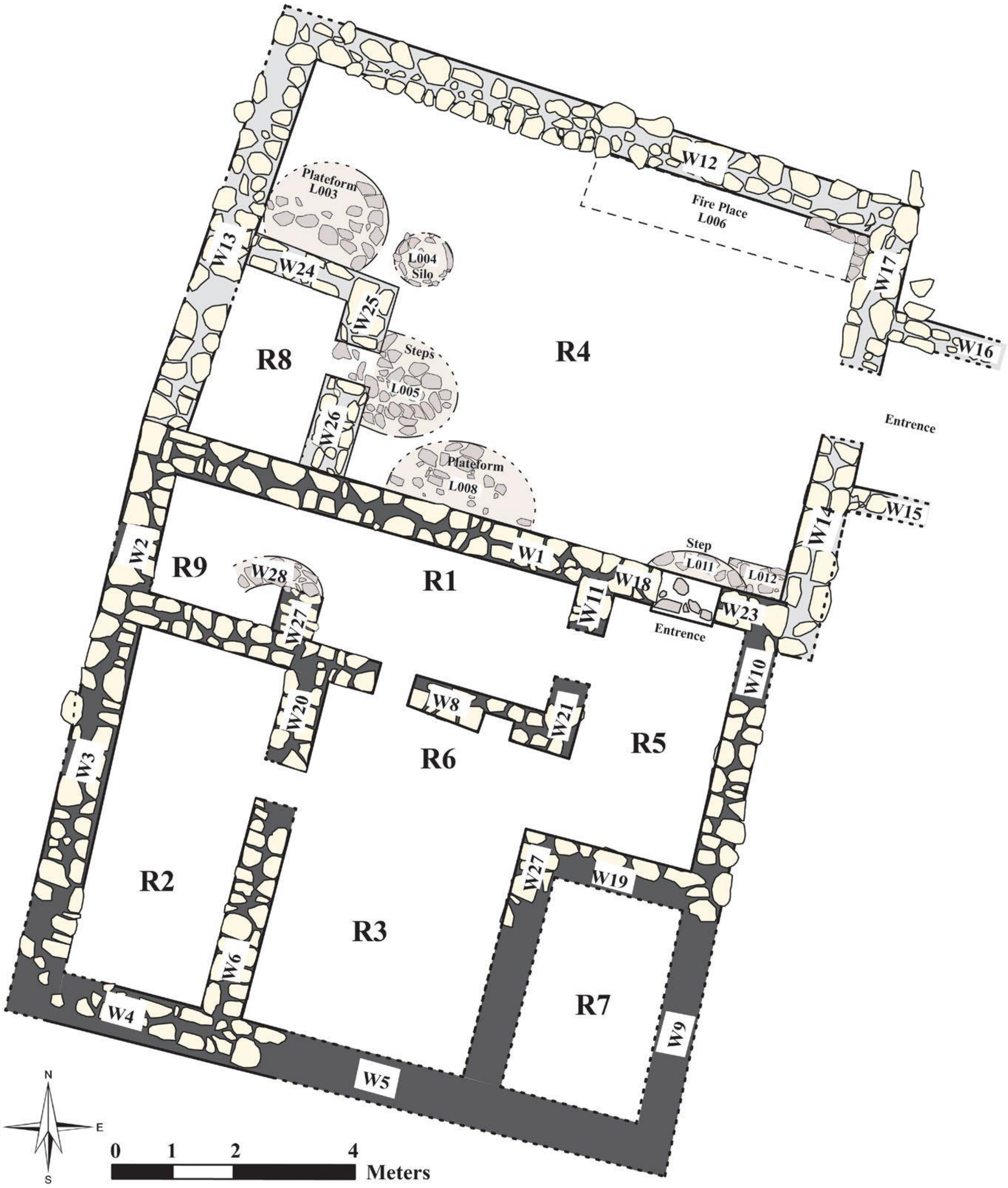

Figure 8: Plan of Building S42 at Dahwa 7. Drawing: M. Hesein, reproduced from [21].

Only a small set of pottery (n=8) from DH7 was sampled for lipid residue analysis. Seven out of the eight samples come from Building S42; one vessel comes from Building S3, a domestic building located on the western part of the settlement. Seven vessels are Sandy Buff ware, which was likely produced locally. Interestingly, some of the rims of Sandy Buff ware found at DH7 resemble shapes of Indus pottery, such as Indus cooking pots [21], of which one example was analysed for lipid residue analysis. The presence of typical Indus shapes among the Dahwa buff sandy ware, such as at Salūt ST-1, has led the scholars to suggest that there may have been Indus potters in the Batinah region or central Oman from *c*. 2500 BC [14, 21]. Other vessels from this ware match known examples of Umm an Nar pottery found at other sites in south-eastern Arabia, such as ridged pots and jars [21]. One of the vessels analysed is an Indus Black-Slipped Jar of Micaceous Red Ware, similar to other examples of potsherds that were produced in the southern Indus basin in Pakistan [e.g. 22]; however, this is still to be confirmed with petrographic and instrumental neutron activation analysis (INAA) of the ceramic paste [21].

1.7. Suwayh 3 (SWY-3)

The site of Suwayh 3, also known as Khor Bani Bu Ali SWY-3, is located 70km south of Ra's al-Hadd, on the eastern coast of Oman. The site lies less than a kilometre away from the present-day coast, and is bounded on its eastern and southern limits by a large sabkha (salt flat). Discovered in 1988 during a survey by members of the Joint Hadd Project in the Ja'alan region, it was first excavated in 1996-1997 [23, 24]. The focus of the excavations was a stone building (Building 1) in the northern part of the site, a test trench (Test trench 1) through and outside Building 1, and a test trench 100 meters from Building 1 (Test trench 2). Several possible walls and hearths were also located on the eastern part of the site during survey, indicating the presence of a settlement[23, 24]. Surface finds included large amounts of

pottery such as sandy red Omani ware, softstone vessels, shell rings, and unusually, a bronze spearhead similar to the types found at sites in the Indus region such as Mohenjo-daro [23].

Excavations of Building 1 revealed a square building with two rectangular rooms located side by side. The architectural plan of Building 1 was similar to other rooms excavated at Ra’s al-Jinz (RJ-2), and technique of construction matches that of Building IV at Ra’s al-Jinz RJ-2 (Period IV, c. 2200-2000 BC), suggesting a contemporary date of occupation at the end of the third millennium BC [23]. Marine shells and fishbones were the only type of faunal remains discovered at the site [23].

Seventeen potsherds from Suwayh 3 were selected for lipid residue analysis. Of these, twelve were from the surface, four were from test trench 1, and a single fragment (SWY3.05: a Mesopotamian sherd) was from test trench 2. Of the seventeen vessels, fifteen were pot/jars of sandy red or sandy red/grey Omani Ware, one was a fine red Umm an-Nar pot/jar, and one was a body fragment of a Mesopotamian pot/jar.

1.8. Kalba 4 (K4)

Kalba is a multi-period site located in the Emirate of Sharjah, on the east coast of United Arab Emirates. With a mound rising 2.5m from the surrounding fields, the site has a considerable depth of preserved stratified archaeological deposits, of which the Early Bronze Age (2500-2000 BC) is most well-known [25]. The site has an Early Bronze Age mudbrick tower around which subsequent architectural developments took place through to the Iron Age.

Kalba 4 lies close to an ancient lagoon which likely had an environment similar to that of today’s: dense mangroves with an abundance of fish, shellfish and birds. The site is also located close to a rich agricultural area. The range of imported pottery, such as Indus and

Mesopotamian pottery, at the site points to its connections with maritime exchange networks [26].

Eleven potsherds from Kalba 4 were selected for lipid residue analysis. The fragments chosen were primarily of Black-Slipped Jars from the Indus region (n=7), to assess the possible contents of these imported vessels; locally-produced Fine Red Ware vessels (n=3); and one fragment that resembled the body or base of a sieve, obtained from the surface.

References

1. Cleuziou S. Excavations at Hili 8: a preliminary report on the 4th to 7th campaigns. Archaeol UAE. 1989;5:61–87.
2. Cleuziou S. The chronology of protohistoric Oman as seen from Hili. In: Costa PM, Tosi M, editors. Oman Studies: Papers in the Archaeology and History of Oman. Rome: Istituto Italiano per il Medio ed Estremo Oriente; p. 47–78. (Serie Orientale Roma, 63).
3. Cleuziou S, Tosi M. In the shadow of the ancestors: The prehistoric foundations of the early Arabian civilization in Oman. Oman: Ministry of Heritage and Culture; 2007.
4. Cleuziou S, Vogt B. Tomb A at Hili North (United Arab Emirates) and its material connections to southeast Iran and the Greater Indus Valley. In: Schotsmans J, Taddei M, editors. South Asian Archaeology 1983. Naples: Istituto Universitario Orientale; 1985. p. 249–77. (Istituto Universitario Orientale Series Minor, 23).
5. Cleuziou S, Méry S, Vogt B. Protohistoire de l’oasis d’Al-Aïn. Travaux de la Mission archéologique française à Abou Dhabi (Émirats Arabes Unis). Les sépultures de l’âge du Bronze. Oxford: Archaeopress; 2011. (British Archaeological Reports, International Series, 2227).

6. Frifelt K. On prehistoric settlement and chronology of the Oman peninsula. East and West. 1975;25:359–424.
7. Frifelt K. A possible link between the Jemdet Nasr and the Umm an-Nar graves of Oman. J Oman Stud. 1975;1:57–80.
8. Munoz O. Pratiques funéraires et paramètres biologiques dans la péninsule d'Oman du Néolithique à la fin de l'âge du Bronze ancien (5–3e millénaires avant notre ère) [PhD thesis]. Paris: Université de Paris 1 Panthéon-Sorbonne/Università di Roma La Sapienza; 2014.
9. Méry S. A funerary assemblage from the Umm an-Nar period: the ceramics from tomb A at Hili North, UAE. Proc Semin Arab Stud. 1997;27:171-91.
10. Blackman MJ, Méry S, Wright RP. Production and exchange of ceramics on the Oman peninsula from the perspective of Hili. J Field Archaeol. 1989;16(1):61–77. doi: https://doi.org.10.1179/jfa.1989.16.1.61
11. Méry S. Les céramiques d'Oman et l'Asie Moyenne: une archéologie des échanges à l'Âge du Bronze. Monographie du Centre de Recherches Archéologiques, 23. Paris: Centre d'études Préhistoire-Antiquité-Moyen Âge; 2000.
12. Degli Esposti M. Excavations at the early bronze age site "ST1" near Bisya (Sultanate of Oman): Notes on the architecture and material culture. In: Stucky RA, Kaelin O, Mathys H-P, editors. Proceedings of the 9th International Congress on the Archaeology of the Ancient Near East. Wiesbaden: Harrassowitz Verlag; 2016. p. 665–678.
13. Frenez D, Degli Esposti M, Méry S, Kenoyer JM. Bronze Age Salut (ST1) and the Indus Civilization: Recent discoveries and new insights on regional interaction. Proc Semin Arab Stud. 2016;46:107-24.

14. Méry S, Degli Esposti M, Frenez D, Kenoyer JM. Indus Potters in Central Oman in the second half of the third millennium BC. First results of a technological and archaeometric study. Proc Semin Arab Stud. 2017;47:163–84.

15. Suryanarayan A, Degli Esposti M, Mery S, Strolin L, Mazuy A, Maraleda-Cibrian N, et al. Domestic food practice and vessel-use at Salūt-ST1, central Oman, during the Umm an-Nar period. Arab Archaeol Epigr. 2024. https://doi.org/10.1111/aae.12247

16. Döpper S. Walk the Line: The 2020 field season of the Al-Mudhaybi Regional Survey. Proc Semin Arab Stud. 2022;51:157-167. doi: https://doi.org/10.1080/0144039X.2022.2040360

17. Swerida J, Cable CM, Dollarhide EN. Survey and Settlement: Preliminary Results of the Bat Archaeological Project's 2019 Field Season. J Oman Stud. 2020;21:82–100.

18. Swerida J, Thornton CP. al-Khafaji reinterpreted: New insights on Umm an-Nar monuments and settlement from Bat, Oman. Arab Archaeol Epigr. 2019;30:1–16. doi: https://doi.org.10.1111/aae.12131

19. Swerida J. Bat and the Umm an-Nar Settlement Tradition. In: Döpper S, editor. Beyond Tombs and Towers: Domestic architecture of the Umm an-Nar period in eastern Arabia. Wiesbaden: Harrassowitz; 2018. p. 51–70.

20. Swerida J, Dollarhide EN, Bryant RC, Mateiciucová I, Přichystal A, Buffington A, et al. Beyond the Oasis: Results from the 2022–23 Field Season of the Bat Archaeological Project. J Oman Stud. In press.

21. Douglas KA, Al-Jahwari NS, Méry S, Hesein M, Williams KD. Umm an-Nar settlement pottery from Dahwa 7 (DH7), northern al-Batinah, Oman. Arab Archaeol Epigr. 2021;31(1):198-212. doi: https://doi.org/10.1111/aae.12263

22. Méry S, Blackman MJ. Harappa et Mohenjo-Daro: deux zones de production de jarres à engobe noir au Pakistan à la période Indus. Paleorient. 1999;25(2):167-77. doi: https://doi.org/10.3406/paleo.1999.4570

23. Méry S, Marquis P. First campaign of excavation at Khor Bani Bu Ali SWY-3, Sultanate of Oman. Proc Semin Arab Stud. 1998;28:215–28.

24. Méry S, Marquis P. Un habitat côtier de l'âge du bronze à Khor Bani Bu Ali SWY-3 (Sultanat d'Oman): deuxième campagne de fouille. Bull Soc Arab Stud. 1999;4:9–12.

25. Eddisford D, Phillips C. Kalbā in the third millennium (Emirate of Sharjah, UAE). Proc Semin Arab Stud. 2009;39:99–112.

26. Eddisford D. Exchange networks of the Early Bronze Age Gulf: The imported ceramics from Kalba 4 (United Arab Emirates). Arab Archaeol Epigr. 2022. https://doi.org/10.1111/aae.12208
