# Supplementary material for: Identifying pastoral and plant products in local and imported pottery in Early Bronze Age southeastern Arabia
Source: PLoS One. 2025 Jun 11;20(6):e0324661. doi: 10.1371/journal.pone.0324661 (PMC12157666; doi:10.1371/journal.pone.0324661)
Supplement: S2 File — (PDF) [file pone.0324661.s002.pdf]

S.I. 2: Details of instrumental analyses

GC-FID analyses

GC-FID analyses were performed for the solvent extracts on an Agilent Technologies 7890A device. One µl of sample was introduced via an on-column injector into a 15 m × 0.32 mm i.d. fused silica capillary (DB5-MS, 0.1 µL film thickness, Agilent J&W), with helium used as the carrier gas. The GC temperature programme was as follows: increased from 50°C to 100°C at 15°C min^{-1}, then from 100°C to 375°C at 10°C min$^{-1.}$

GC-MS analyses

GC-MS analysis of both solvent and acidified methanol extracts were performed on a Shimadzu GC 2010 PLUS chromatograph coupled to a Shimadzu QP 2010 ULTRA mass spectrometer, fitted with a high temperature nonpolar column (DB5-HT, 15 m × 0.322 mm i.d., 0.1 µm film thickness, Agilent J&W). The injection was performed using a splitless injector. The temperature programme consisted of a 1 min isothermal hold at 50°C followed by an increase to 150°C at 20°C·min^{-1}, then to 250°C at 10°C·min^{-1} and to 350°C·min^{-1} and a final isothermal hold for 10 min. The GC–MS interface was maintained at a temperature of 300°C and the mass spectrometer run in electron ionization mode (EI, 70 eV). Mass spectra were acquired over the range *m/z* 50–950.

Molecular compounds were identified on the basis of their characteristic ions. For the acidified methanol extracts, these included fatty acids (*m/z* 74, 87), dicarboxylic acids(*m/z* 55, 98, 152), *n*-alkanes (*m/z* 57, 71, 85), ketones (*m/z* 59, 211, 227, 239, 255, 267). For trimelthylsilylated samples, fatty acids (*m/z* 132), *n*-alcohols (*m/z* 103), *n*-alkanes (*m/z* 85), cholesterol derivates (*m/z* 369), sterols (*m/z* 213), mono-, di- and triacylglycerols (*m/z* 129) and esters (*m/z* 257, 285) were identified.

GC-C-IRMS analyses

Fifty-one samples from specific vessel types (e.g. BSJs) and with relatively high concentrations of fatty acids were selected from Hili 8 (n = 11), Salut ST1 (n = 19), Bat (Settlement Slope, n = 9), Mukhtru (n=5) and Kalba 4 (n = 7). Stable carbon isotope values of methyl palmitate ($C_{16:0}$) and methyl stearate ($C_{18:0}$), derived from precursor fatty acids, were measured by GC-C-IRMS following existing procedures [1].

Stable carbon isotopic compositions of $C_{16:0}$ and $C_{18:0}$ fatty acids were determined using a Delta V Advantage mass spectrometer (Thermo Fisher, Bremen) linked to a Trace Ultra GC with a ConFlo IV interface. Samples solved in isooctane were injected in splitless mode on a J&W Scientific DB5-MS UI fused silica column (60 m x 0.25mm i.d., 0.25 μm film thickness). The effluent from the GC passed from the GC column immediately into and through a combustion reactor consisting of a NiO tube, CuO, NiO and Pt wires which was held at 1000 °C. The effluent then passed through a water separator consisting of a Nafion tube prior to entering the MS. The GC programme was ramped from 90 °C (1 min) to 320 °C at 6 °C/min and held at 320 °C for 20 minutes. The injector was held at 310 °C. Helium was used as the carrier gas at a ramped flow, it was held at 2 mL/min for 1 min and ramped to -0.02 mL/min^2 to 1.2 mL/min and held for 19 min. The effluent from the GC was diverted away from the combustion reactor during the initial period of solvent elution and out of a divert valve to the atmosphere (backflush mode), while helium was passed backwards through the combustion reactor. During the solvent–divert period, CO_2 reference gas was automatically introduced into the isotope ratio mass spectrometer in a series of pulses and its $^{13}C/^{12}C$ ratios measured. After the solvent-divert period, the effluent from the GC was allowed to enter the combustion reactor and IRMS. The IRMS automatically measured the ion intensities of *m/z* 44, 45, 46 in its three Faraday cups corresponding to $^{12}C^{16}O_2$, $^{13}C^{16}O_2$, and $^{12}C^{16}O^{18}O$ respectively. The Isodat 3.0 software automatically computed the $^{13}C/^{12}C$ of

each sample peak, referenced to the standard CO_2 gas and its known $^{13}C/^{12}C$ content. The results were presented in per mil (‰) relative to VPDB standard. To ensure data comparability, the instrument was calibrated using the Indiana University F8 standard. This standard contains a mixture of 8 methylated and ethylated fatty acids of known isotopic composition. Additionally, quality control standards were intercalated every 5-6 injections to control the stability of the isotopic measurements over time. Instrumental precision and accuracy were better than ± 0.5‰. Over half of the lipid extracts were analysed in duplicate to ensure that the instrumental reproducibility and accuracy was also achieved with sample extracts. The averaged distance between replicates applied to sample extracts was 0.05 and 0.01‰ for $C_{16:0}$ and $C_{18:0}$, respectively. Finally, the measured isotopic values for FAMEs were corrected to account for methylation through comparisons with a standard of $C_{16:0}$ and $C_{18:0}$ fatty acids of known isotopic composition that were processed with each batch under identical conditions. Results were plotted against $\delta^{13}C$ values of modern reference terrestrial fats from Africa and Asia and C_3 plant oil references published elsewhere [2-8], as fatty acid $\delta^{13}C$ values of modern references fats from the study region are not yet available.

References

1. O. E. Craig, R. B. Allen, A. Thompson, R. E. Stevens, V. J. Steele, C. Heron. Distinguishing wild ruminant lipids by gas chromatography/combustion/isotope ratio mass spectrometry. Rapid Commun. Mass Spectrom. 2012, 26, 2359. doi: https://doi.org/10.1002/rcm.6349
2. Craig OE, Chapman J, Heron CP, Willis LH, Bartosiewicz L, Taylor G, Whittle A, Collins MJ. Did the first farmers of central and eastern Europe produce dairy foods? Antiquity. 2005;79(306):882–894. doi: https://doi.org/10.1017/S0003598X00115017

3. Spangenberg JE, Jacomet S, Schibler J. Chemical analyses of organic residues in archaeological pottery from Arbon Bleiche 3, Switzerland- evidence for dairying in the late Neolithic. J Archaeol Sci. 2006;33(1):1–13. doi: https://doi.org/10.1016/j.jas.2005.05.013

4. Outram AK, Stear NA, Bendrey R, Olsen S, Kasparov A, Zaibert V, Thorpe N, Evershed RP. The earliest horse harnessing and milking. Science. 2009;323(5919):1332-1335. doi: https://doi.org/10.1126/science.1168594

5. Gregg MW, Banning EB, Gibbs K, Slater GF. Subsistence practices and pottery use in Neolithic Jordan: molecular and isotopic evidence. J Archaeol Sci. 2009;36(4):937–946. doi: https://doi.org/10.1016/j.jas.2008.09.009

6. Lucquin A, Gibbs K, Uchiyama J, Saul H, Ajimoto M, Eley Y, Radini A, Heron CP, Shoda S, Nishida Y, Lundy J, Jordan P, Isaksson S, Craig OE. Ancient lipids document continuity in the use of early hunter-gatherer pottery through 9,000 years of Japanese prehistory. Proc Natl Acad Sci USA. 2016;113(15):3991–3996. doi: https://doi.org/10.1073/pnas.1522908113

7. Dunne J, Evershed RP, Salque M, Cramp L, Bruni S, Ryan K, Biagetti S, Lernia S. First dairying in green Saharan Africa in the fifth millennium BC. Nature. 2012;486(7403):390–394. doi: https://doi.org/10.1038/nature11186

8. Steele VJ, Stern B, Stott AW. Olive oil or lard?: distinguishing plant oils from animal fats in the archaeological record of the eastern Mediterranean using gas chromatography/combustion/isotope ratio mass spectrometry. Rapid Commun Mass Spectrom. 2010;24(23):3478–3484. doi: https://doi.org/10.1002/rcm.4790
