# Supplementary material for: Identifying pastoral and plant products in local and imported pottery in Early Bronze Age southeastern Arabia
Source: PLoS One. 2025 Jun 11;20(6):e0324661. doi: 10.1371/journal.pone.0324661 (PMC12157666; doi:10.1371/journal.pone.0324661)
Supplement: S3 File — (PDF) [file pone.0324661.s003.pdf]

S3_R code for figures and statistical analysis

Akshyeta Suryanarayan

2024-09-04

R Markdown

This is an R Markdown document. Markdown is a simple formatting syntax for authoring HTML, PDF, and MS Word documents. For more details on using R Markdown see http://rmarkdown.rstudio.com.

##This document contains the R code and statistics for Suryanarayan et al.,'Identifying pastoral and plant products in local and imported pottery in Early Bronze Age southeastern Arabia'.

##Install and load libraries

```
library(readr)
library(readxl)
library(ade4)
library(ggplot2)
library(dplyr)
```

```
##
## Attaching package: 'dplyr'

## The following objects are masked from 'package:stats':
##
##     filter, lag

## The following objects are masked from 'package:base':
##
##     intersect, setdiff, setequal, union
```

```
library(ggpubr)
library(reshape2)
library(RColorBrewer)
library(httpuv)
library(caTools)
library(tidyr)
```

```
##
## Attaching package: 'tidyr'

## The following object is masked from 'package:reshape2':
##
##     smiths
```

```r
library(viridis)
```

```
## Loading required package: viridisLite
```

###Download data file, change " "C:/Users/franc/Dropbox/R" to the name of the folder where you have saved the data files #Load data

```r
arabia <- read.csv("C:/Users/franc/Dropbox//R/S4.csv")
arabia <- droplevels(arabia)
```

#DATA PREPARATION #Convert variables to factors

```r
arabia$Sample_ID <- factor(arabia$Sample_ID)
arabia$Vessel_origin <- factor(arabia$Vessel_origin)
arabia$Site_name <- factor(arabia$Site_name)
arabia$Context_or_ID <- factor(arabia$Context_or_ID)
arabia$Rim_base_body <- factor(arabia$Rim_base_body)
arabia$Vessel_form <- factor(arabia$Vessel_form)
arabia$Type <- factor(arabia$Type)
arabia$Sub_type <- factor(arabia$Sub_type)
arabia$Cultural_period <- factor(arabia$Cultural_period)
arabia$Period <- factor(arabia$Period)
arabia$Period_grouping <- factor(arabia$Period_grouping)
```

#Exclude samples with lipid concentrations below 5

```r
dt <- subset (arabia,(Site_name == "Hili 8" | Site_name == "Hili North Tomb A"|
                        Site_name == "Bat"|
                        Site_name == "Salut ST1" | Site_name == "Mukhtru" | Site_name == "Dahwa 7"
                      | Site_name == "Kalba 4" | Site_name == "Suwayh 3"))
dt <- droplevels(dt)


d <- subset(dt, Lipid_concentration >= 5)
```

#subset for Early Bronze Age periods

```r
eba <- droplevels(subset (d, Cultural_period == "UAN" |
                            Cultural_period == "Hafit"|
                            Cultural_period == "Wadi Suq"))
eba$Site_name <- factor(eba$Site_name, levels = c('Hili 8',
                                                  'Hili North Tomb A',
                                                  'Bat','Salut ST1','Mukhtru',
                                                  'Dahwa 7', 'Kalba 4',
                                                  'Suwayh 3'))
```

#subset for vessel origin

```r
origin <- droplevels(subset (eba,(Vessel_origin == "Local" |
                                    Vessel_origin == "Regional"|
                                    Vessel_origin == "Mesopotamian" |
                                    Vessel_origin == "Indus"|
                                    Vessel_origin == "Makran" |
                                    Vessel_origin == "Local-Makran imitation"|
                                    Vessel_origin == "Unknown")))
origin$Vessel_origin <- factor(origin$Vessel_origin, levels = c('Local',
                                                               'Regional',
                                                               'Local-Makran imitation',
                                                               'Mesopotamian',
                                                               'Makran',
                                                               'Indus',
                                                               'Unknown'))
```

#exclude vessel from surface contexts

```r
vessels <- subset (d,(!Context_or_ID == "surface"))
vessels <- subset (vessels, (!Additional_contextual_info == "surface"))
```

#subset Sandy Wares (local), Fine Red Wares (regionally distributed) and Indus BSJs

```r
SW <- subset (eba,Vessel_origin == "Local")
FROM <- subset (eba,Vessel_origin == "Regional")
BSJ <- subset (eba, Vessel_form == "BSJ")
```

#subset inland and coastal sites

```r
inland <- subset (eba,(Site_name == "Hili 8" | Site_name == "Hili North Tomb A" | Site_name == "Bat"|
                            Site_name == "Salut ST1" | Site_name == "Mukhtru" | Site_name == "Dahwa 7")
inland <- droplevels(inland)
coastal <- subset (eba,(Site_name == "Kalba 4" | Site_name == "Suwayh 3" ))
coastal <- droplevels(coastal)
```

#arrange order in which site names and vessels appear

```r
eba$Site_name <- factor(eba$Site_name, levels = c('Hili 8', 'Hili North Tomb A',
                                                  'Bat','Salut ST1','Mukhtru',
                                                  'Dahwa 7', 'Kalba 4',
                                                  'Suwayh 3'))
inland$Site_name <- factor(inland$Site_name, levels = c('Hili 8',
                                                        'Hili North Tomb A',
                                                        'Bat','Salut ST1',
                                                        'Mukhtru', 'Dahwa 7'))
eba$Vessel_origin <- factor(eba$Vessel_origin, levels = c('Local', 'Regional',
                                                          'Local-Makran imitation',
                                                          'Mesopotamian',
                                                          'Makran',
                                                          'Indus', 'Unknown'))
```

##FIGURE 2

#FIGURE 2A #Histogram of lipid concentration of all EBA pottery (excluding surface pottery), on log scale

```
fig2a <- ggplot(eba, aes(x= Lipid_concentration)) +
  geom_histogram(aes(y=..density..), colour="black", fill="white", bins = 20)+
  geom_density(alpha=.2, fill="#1f78b4") +
  geom_vline(aes(xintercept=mean(Lipid_concentration)),
                           color="black", linetype="dashed", size=1) +
  scale_x_log10(breaks = c(5, 10, 20, 50, 100, 200, 300, 500, 1000, 2000, 5000)) +
  labs(x = "log(Lipid concentration in µg.g-1)") +
  theme_classic(base_size = 10) +  coord_flip()
```

```
## Warning: Using `size` aesthetic for lines was deprecated in ggplot2 3.4.0.
## i Please use `linewidth` instead.
## This warning is displayed once every 8 hours.
## Call `lifecycle::last_lifecycle_warnings()` to see where this warning was
## generated.
```

```
fig2a
```

```
## Warning: The dot-dot notation (`..density..`) was deprecated in ggplot2 3.4.0.
## i Please use `after_stat(density)` instead.
## This warning is displayed once every 8 hours.
## Call `lifecycle::last_lifecycle_warnings()` to see where this warning was
## generated.
```

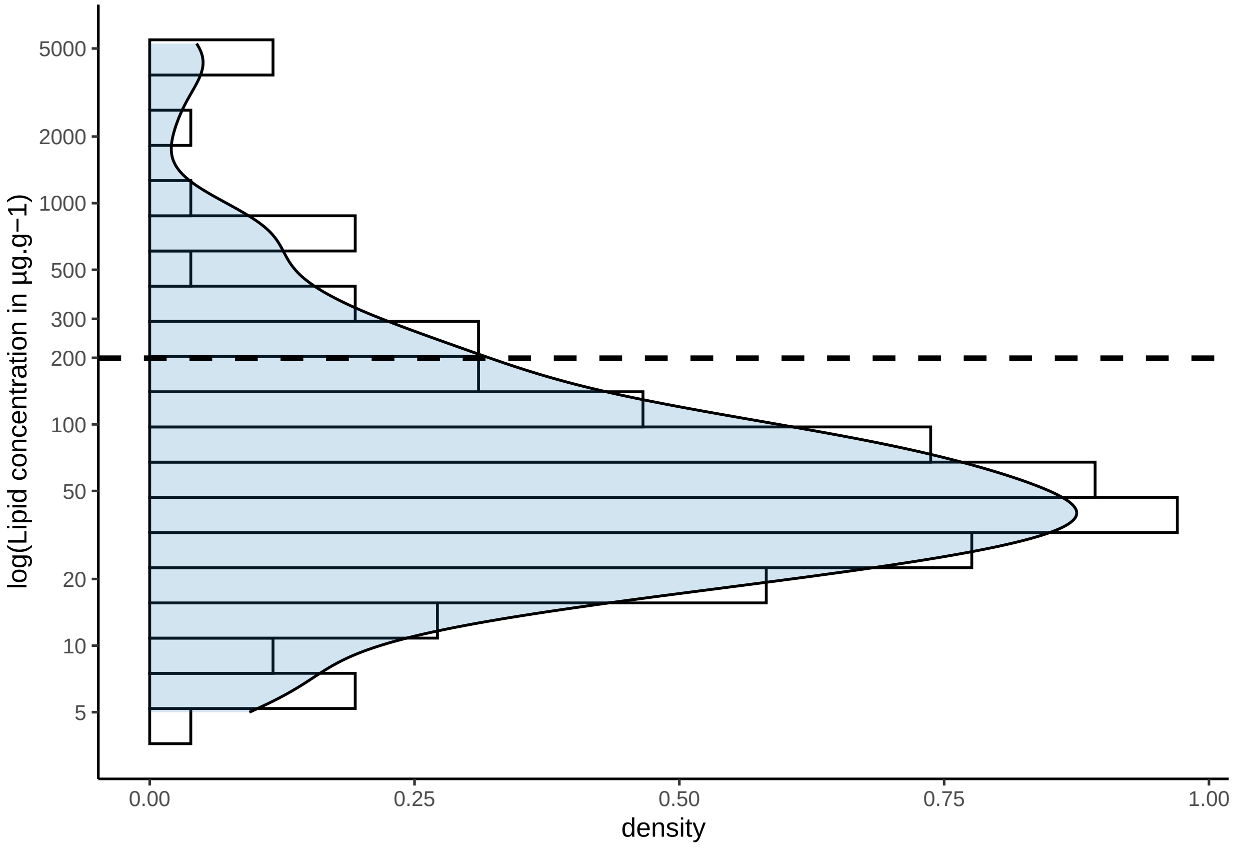

#FIGURE 2B #Boxplots of lipid concentration of pottery by site, with vessel origins marked in unique colours

```
fig2b <- ggplot(data=eba,
       mapping=aes(x=Site_name,y=Lipid_concentration)) +
  geom_boxplot(outlier.shape = NA) +
  geom_point(shape = 17, size = 2.5, position = position_jitterdodge(),mapping=aes(color=Vessel_origin)
scale_x_discrete(labels = paste(levels(eba$Site_name),"\n(N=",table(eba$Site_name),")",sep="")) +
  scale_y_log10(breaks = c(5, 10, 20, 50, 100, 200, 300, 500, 1000, 2000, 5000)) +
  scale_colour_manual(values = c("#E69F00", "#1b9e77", "cyan","#984ea3", "grey", "#000000", "red")) +
  labs(x = "Site name",
       y = "log(Lipid concentration in µg.g-1)", colour = "Vessel origin") +
  theme_classic(base_size = 10) + theme(axis.text.x=element_text(angle=90,hjust=1,vjust=0.5))
fig2b
```

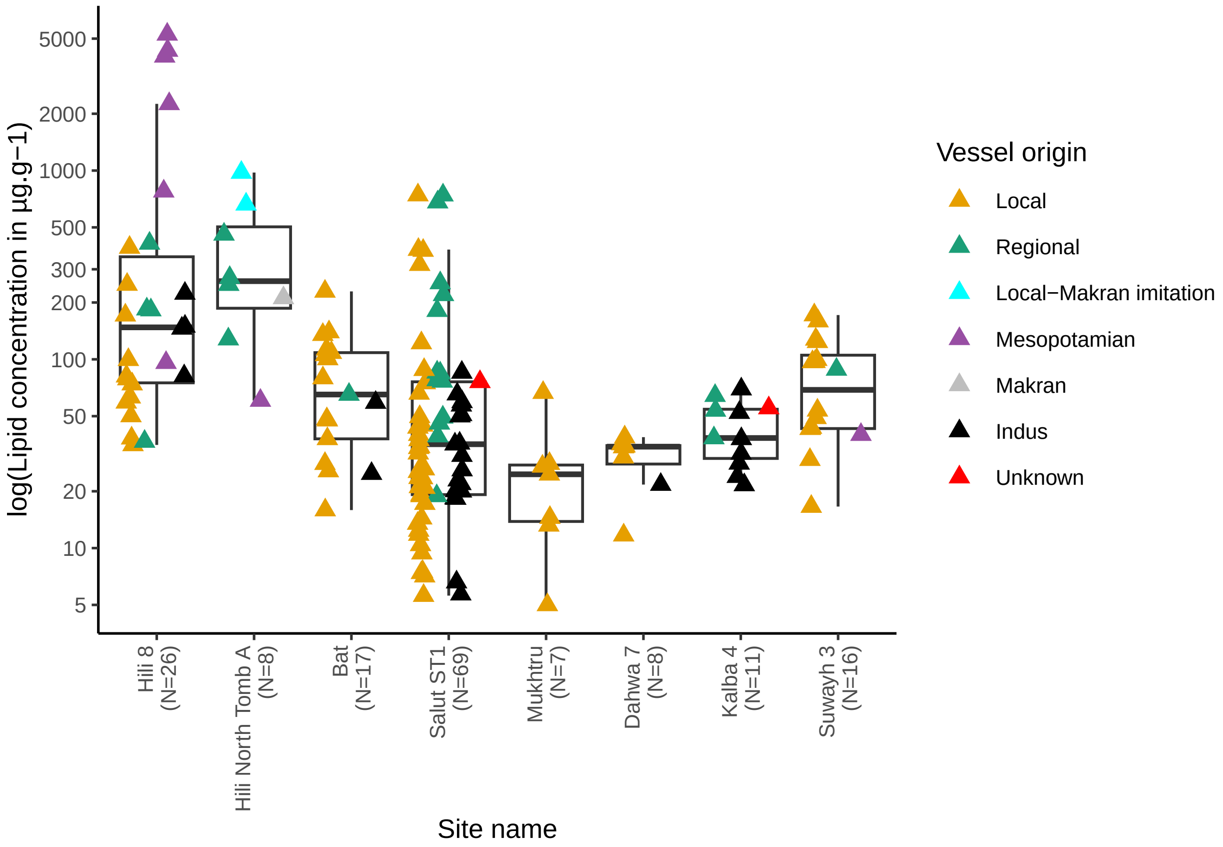

#STATISTICS #MEAN AND MEDIAN LIPID CONCENTRATIONS OF LOCAL, REGIONAL and IMPORTED VESSELS

```
mean(BSJ$Lipid_concentration)
```

```
## [1] 50.025
```

```
median(BSJ$Lipid_concentration)
```

```
## [1] 33.6
```

```r
mean(SW$Lipid_concentration)
```

```
## [1] 77.37111
```

```r
median(SW$Lipid_concentration)
```

```
## [1] 39.3
```

```r
mean(FROM$Lipid_concentration)
```

```
## [1] 180.037
```

```r
median(FROM$Lipid_concentration)
```

```
## [1] 85.4
```

#Lipid concentration on log scale

```r
vessels$lipid_log <- log10(vessels$Lipid_concentration)
```

#Normality check

```r
ggqqplot(vessels$lipid_log, ylab = "lipid concentration in ug/g")
```

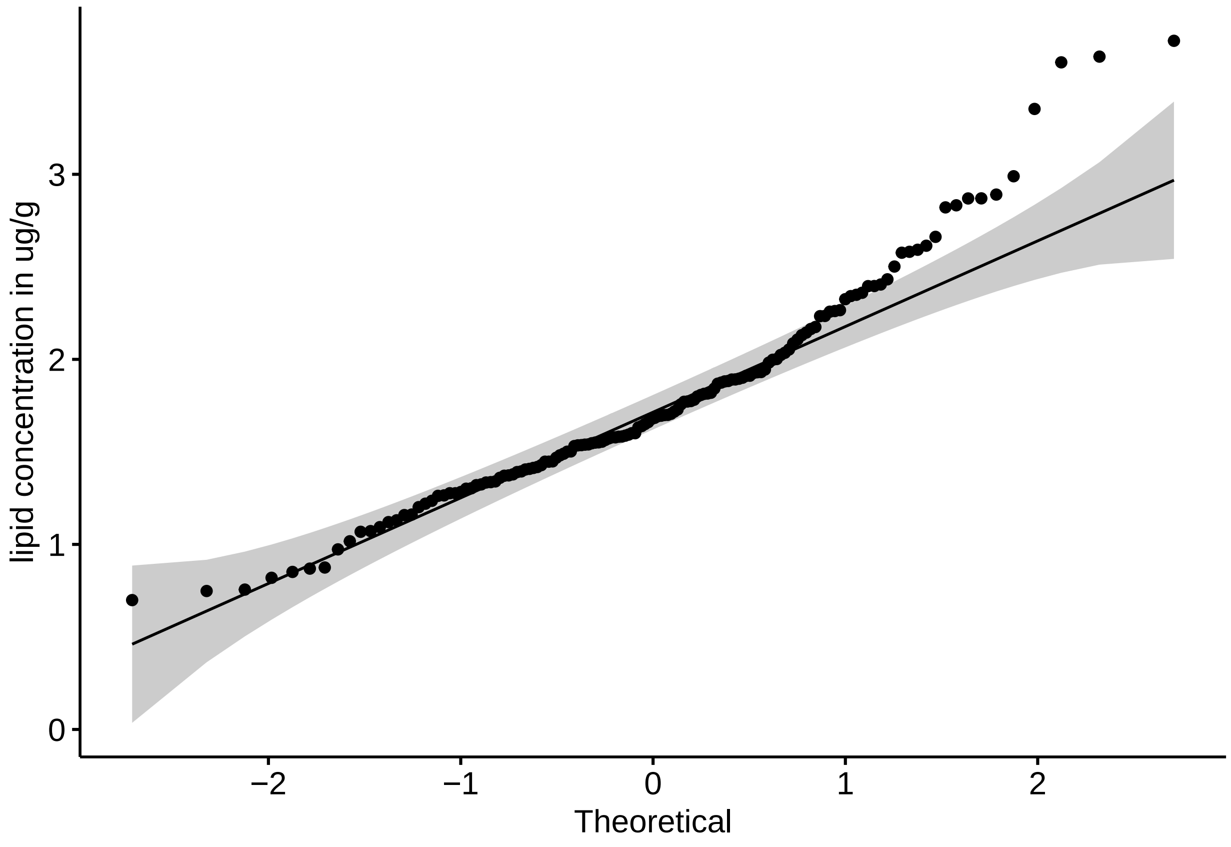

```r
#data is not normal
```

#Kruskal-Wallis test for effect of site on lipid concentrations

```r
kruskal.test(lipid_log~Vessel_origin,data= vessels)
```

```
##
##  Kruskal-Wallis rank sum test
##
## data:  lipid_log by Vessel_origin
## Kruskal-Wallis chi-squared = 38.209, df = 6, p-value = 1.022e-06
```

```r
#test supports difference between groups
```

##Which pairs of groups are different? #Pairwise Wilcox test

```r
pairwise.wilcox.test(vessels$lipid_log, vessels$Vessel_origin,
                     p.adjust.method = "BH")
```

```
##
##  Pairwise comparisons using Wilcoxon rank sum test with continuity correction
##
## data:  vessels$lipid_log and vessels$Vessel_origin
##
##                         Indus   Local   Local-Makran imitation Makran
## Local                   0.86579 -       -                      -
## Local-Makran imitation 0.01497 0.06349 -                      -
## Makran                  0.28283 0.35104 0.86579                -
## Mesopotamian            0.00073 0.00236 0.93333                0.93333
## Regional                0.00023 0.00031 0.06349                0.86579
## Unknown                 0.69421 0.77494 0.86579                1.00000
##                         Mesopotamian Regional
## Local                   -            -
## Local-Makran imitation -            -
## Makran                  -            -
## Mesopotamian            -            -
## Regional                0.10118      -
## Unknown                 0.86579      0.86579
##
## P value adjustment method: BH
```

```r
#test supports differences between several sites
```

#FIGURE 3 #Prepare data ##Subset isotopic data

```r
vessels$Period_grouping <- factor(vessels$Period_grouping, levels = c('Hafit-Early UAN', 'Mid UAN', 'La
iso <- subset (vessels,(!is.na(vessels$d13C16)))
```

#subset Umm an-Nar pottery data

```
uan <- droplevels(subset (vessels, Cultural_period == "UAN"))
iso_uan <- subset (uan,(!is.na(uan$d13C16)))
iso_uan$Vessel_origin <- factor(iso_uan$Vessel_origin, levels = c('Local', 'Regional', 'Indus'))
```

#subset Umm an-Nar pottery data


```
uan <- droplevels(subset (vessels, Cultural_period == "UAN"))
iso_uan <- subset (uan,(!is.na(uan$d13C16)))
iso_uan$Vessel_origin <- factor(iso_uan$Vessel_origin, levels = c('Local', 'Regional', 'Indus'))
```


#subset Hafit and early Umm an-Nar pottery data

```
hafit <- droplevels(subset (iso, Period_grouping == "Hafit-Early UAN"))
```

#ADD REFERENCE DATA #Download data file, change " "C:/Users/franc/Dropbox/R" to the name of the folder where you have saved the data files

```
data.refs1 <- read.csv("C:/Users/franc/Dropbox/R/reference-fats-nonrum-rum-dairy.csv")
data.refs2 <- read.csv("C:/Users/franc/Dropbox/R/PhD/Data/references_big_delta.csv") #this files includ
```

#Create summaries of reference data, this will be needed to generate Figures 4b and 5b

```
ref.summary1 <- data.refs1 %>%
  group_by(Fat_source) %>%
  summarise(
    sd = sd(bigdelta, na.rm = TRUE),
    bigdelta = mean(bigdelta))
ref.summary1
```

```
## # A tibble: 3 x 3
##   Fat_source          sd bigdelta
##   <chr>            <dbl>    <dbl>
## 1 Non-ruminant      1.14     1.33
## 2 Ruminant adipose  1.06    -1.55
## 3 Ruminant dairy    1.41    -4.77
```

```
ref.summary2 <- data.refs2 %>%
  group_by(Fat_source) %>%
  summarise(
    sd = sd(bigdelta, na.rm = TRUE),
    bigdelta = mean(bigdelta))
ref.summary2
```

```
## # A tibble: 4 x 3
##   Fat_source          sd bigdelta
##   <chr>            <dbl>    <dbl>
## 1 C3 plant oil     0.847   -0.262
## 2 Non-ruminant     1.14     1.33
## 3 Ruminant adipose 1.06    -1.55
## 4 Ruminant dairy   1.41    -4.77
```

#FIGURE 4

#Figure 4a

```
fig4a <- ggplot() + geom_point (size = 3, data = hafit, aes (x = d13C16, y = d13C18, colour = Vessel_ori
  geom_abline(intercept = -3.1, col='black', linetype = "dashed") +
  geom_abline(intercept = 0, col='grey') +
  expand_limits(x = c(-16,-33), y = c(-16, -33)) +
  scale_y_continuous(breaks=c(-32, -28, -24,-20, -16)) +
  scale_x_continuous(breaks=c(-32, -28, -24,-20, -16)) +
  theme(plot.title = element_text(hjust=0, size=16)) +
  labs(x = expression(delta^13* C [16:0]* " "("\u2030")),
       y = expression(delta^13* C [18:0]* " "("\u2030")),  color = "Vessel origin") +
  scale_colour_manual(values = c("#E69F00", "#984ea3")) +
  theme_classic()
fig4a
```

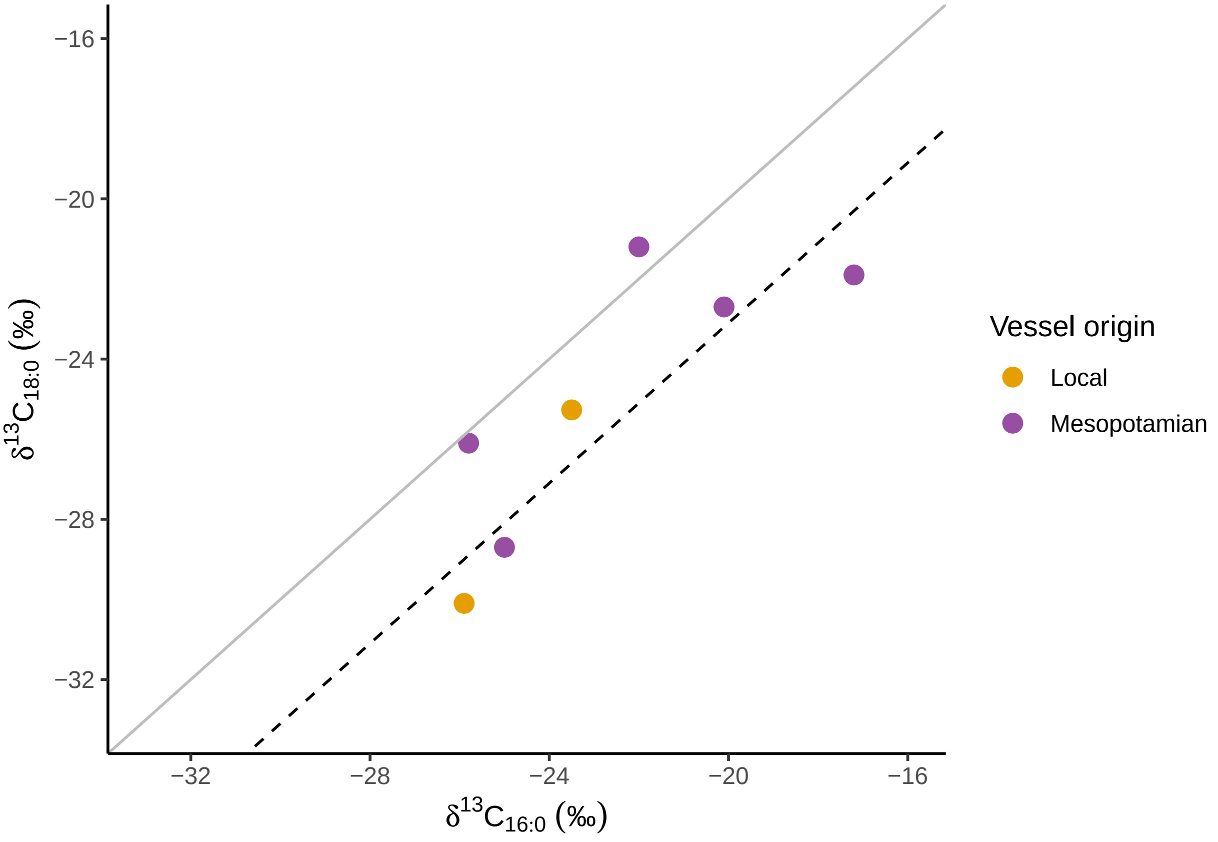

#Figure 4b

```
fig4b <- ggplot() + geom_point (size = 2.8, data = hafit, aes (x = d13C16, y = bigdelta, colour = Vessel
  geom_pointrange (data = ref.summary1,  aes(x = c(-31.7, -31.7, -31.7) , y = bigdelta, ymin = bigdelta-
  theme(plot.title = element_text(hjust=0, size=16)) +
  expand_limits(x = c(-16,-33), y = c(-6, 3)) +
  geom_segment(x = -27, y = 3.8, xend = -20, yend = 3.8, size = 0.3,
               arrow = arrow(length = unit(0.3, "cm")), colour = "black", inherit.aes = FALSE) +
  scale_y_continuous(breaks=c(-6, -4, -2, 0, 2)) +
```

```
  scale_x_continuous(breaks=c(-32, -28, -24,-20, -16)) +
  scale_colour_manual(values = c("#E69F00", "#984ea3")) +
    annotate("text", x=-32.4, y=-4.8, label= "Ruminant Dairy", size=3.2, angle=90) +
    annotate("text", x=-32.4, y=-1.5, label= "Ruminant Adipose", size=3.2, angle=90) +
    annotate("text", x=-32.4, y=1.5, label= "Non-ruminant", size=3.2, angle=90) +
    #annotate("text", x=-31.4, y= -0.2, label= "Plant oil", size=3.2, angle=90) +
    annotate("text", x=-30, y=3.2, label= "C3-input", size=3.2, angle=0)+
    annotate("text", x=-22, y=3.2, label= "Increasing C4/marine input", size=3.2, angle=0)+
  labs(x = expression(delta^13* C [16:0]* " "("\u2030")),
       y = expression(Delta^13* C * " "("\u2030")), colour = "Vessel origin") + theme_classic()
fig4b
```

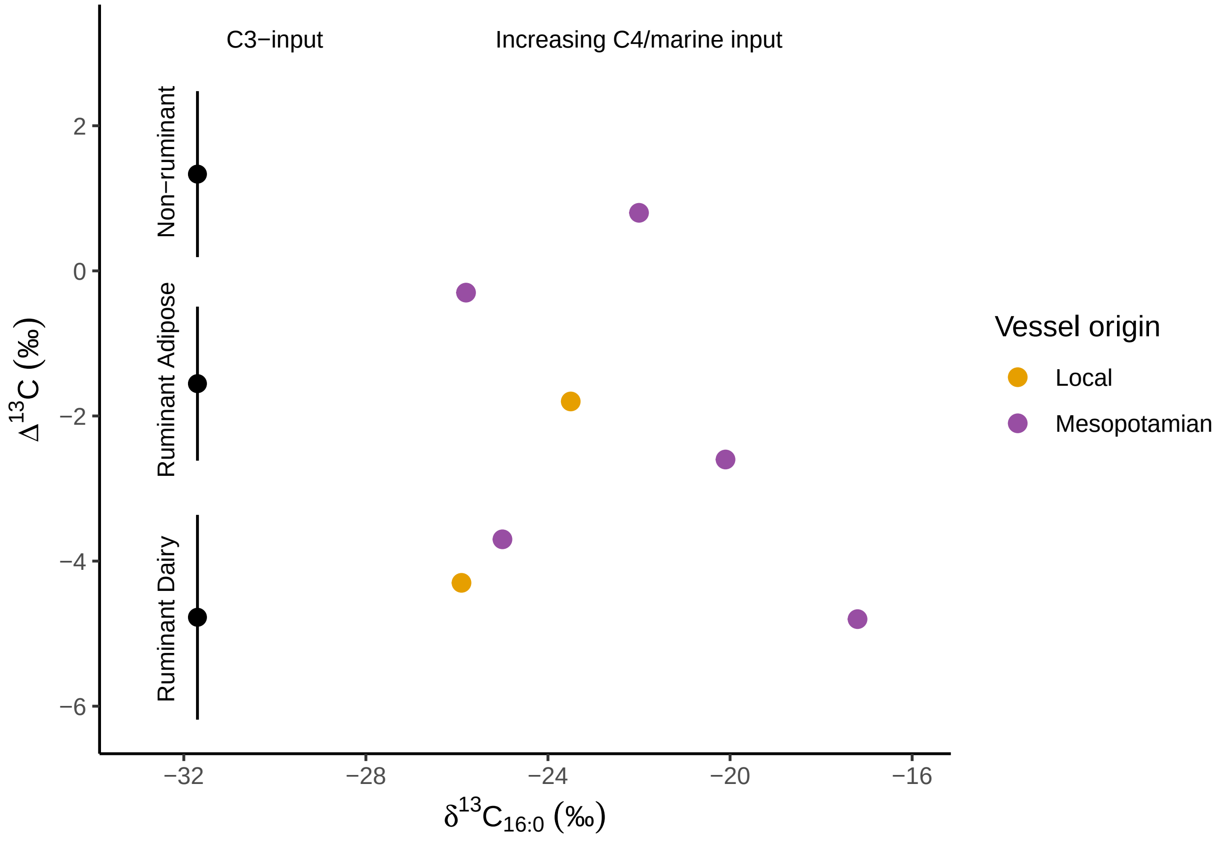

#FIGURE 5

#Figure 5a

```
fig5a <- ggplot() + geom_point (size = 3, data = iso_uan, aes (x = d13C16, y = d13C18, colour = Vessel_o
  #stat_ellipse(type = "norm", data = data.refs1, aes (x= d13C_C16, y = d13C_C18, fill = Fat_source), g
  geom_abline(intercept = -3.1, col='black', linetype = "dashed") +
  geom_abline(intercept = 0, col='grey') +
  expand_limits(x = c(-16,-33), y = c(-16, -33)) +
  scale_y_continuous(breaks=c(-32, -28, -24,-20, -16)) +
  scale_x_continuous(breaks=c(-32, -28, -24,-20, -16)) +
  theme(plot.title = element_text(hjust=0, size=16)) +
  labs(x = expression(delta^13* C [16:0]* " "("\u2030")),
       y = expression(delta^13* C [18:0]* " "("\u2030")),  color = "Vessel origin", shape = "Site name")
```

```
  scale_shape_manual(values = c(3, 1, 2, 10, 7)) +
  scale_colour_manual(values = c("#E69F00", "#1b9e77", "#000000")) +
  theme_classic()
fig5a
```

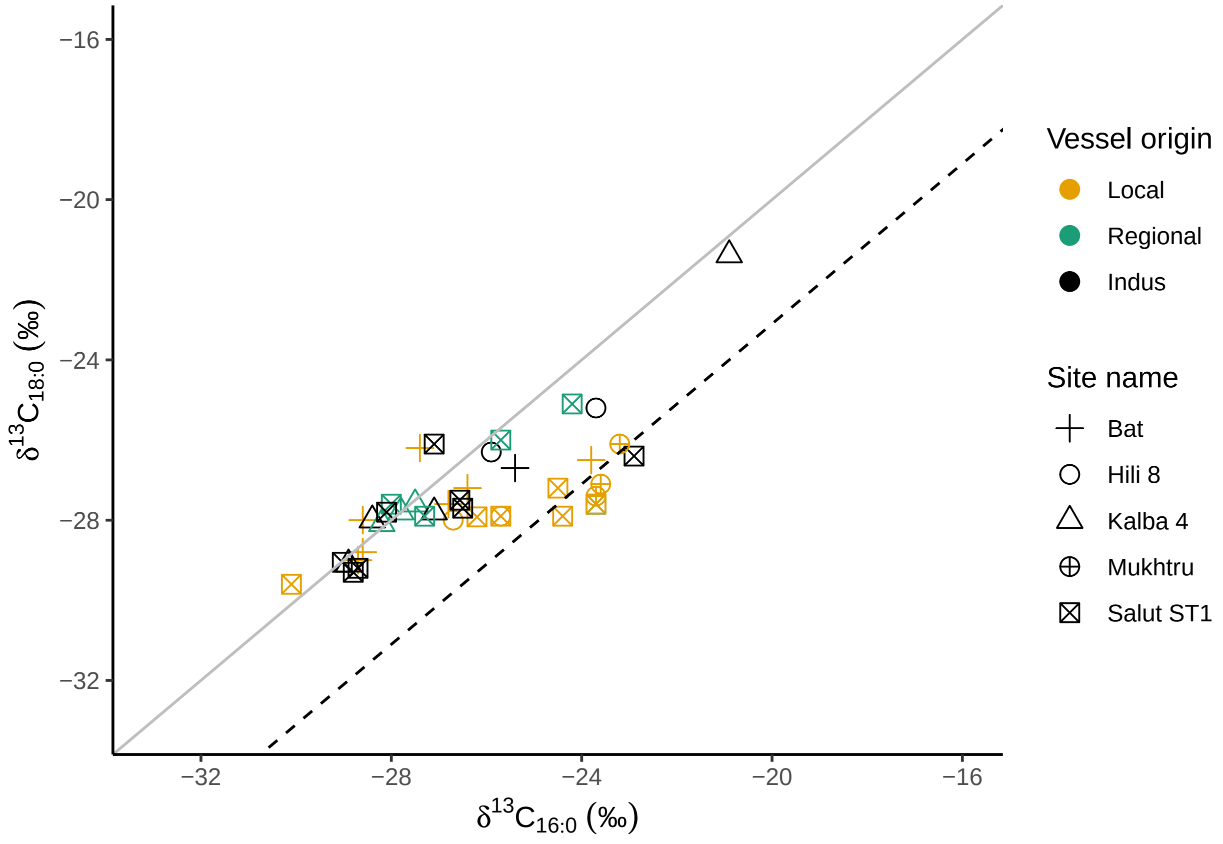

#Figure 5b

```
fig5b <- ggplot() + geom_point (size = 2.8, data = iso_uan, aes (x = d13C16, y = bigdelta, shape = Site_
  geom_pointrange (data = ref.summary2,  aes(x = c(-30.9, -31.7, -31.7, -31.7) , y = bigdelta, ymin = bi
  theme(plot.title = element_text(hjust=0, size=16)) +
  expand_limits(x = c(-16,-33), y = c(-6, 3)) +
  geom_segment(x = -27, y = 3.8, xend = -20, yend = 3.8, size = 0.3,
               arrow = arrow(length = unit(0.3, "cm")), colour = "black", inherit.aes = FALSE) +
  scale_y_continuous(breaks=c(-6, -4, -2, 0, 2, 4)) +
  scale_x_continuous(breaks=c(-32, -28, -24,-20, -16)) +
  scale_shape_manual(values = c(3, 1, 2, 10, 7)) +
 scale_colour_manual(values = c("#E69F00", "#1b9e77", "#000000")) +
  annotate("text", x=-32.4, y=-4.8, label= "Ruminant Dairy", size=3.2, angle=90) +
  annotate("text", x=-32.4, y=-1.5, label= "Ruminant Adipose", size=3.2, angle=90) +
  annotate("text", x=-32.4, y=1.5, label= "Non-ruminant", size=3.2, angle=90) +
  annotate("text", x=-31.4, y= -0.2, label= "Plant oil", size=3.2, angle=90) +
  annotate("text", x=-29.9, y=3.2, label= "C3-input", size=3.2, angle=0)+
  annotate("text", x=-22, y=3.2, label= "Increasing C4/marine input", size=3.2, angle=0)+
  labs(x = expression(delta^13* C [16:0]* " "("\u2030")),
       y = expression(Delta^13* C * " "("\u2030")), colour = "Vessel origin", shape = "Site name") + the
fig5b
```

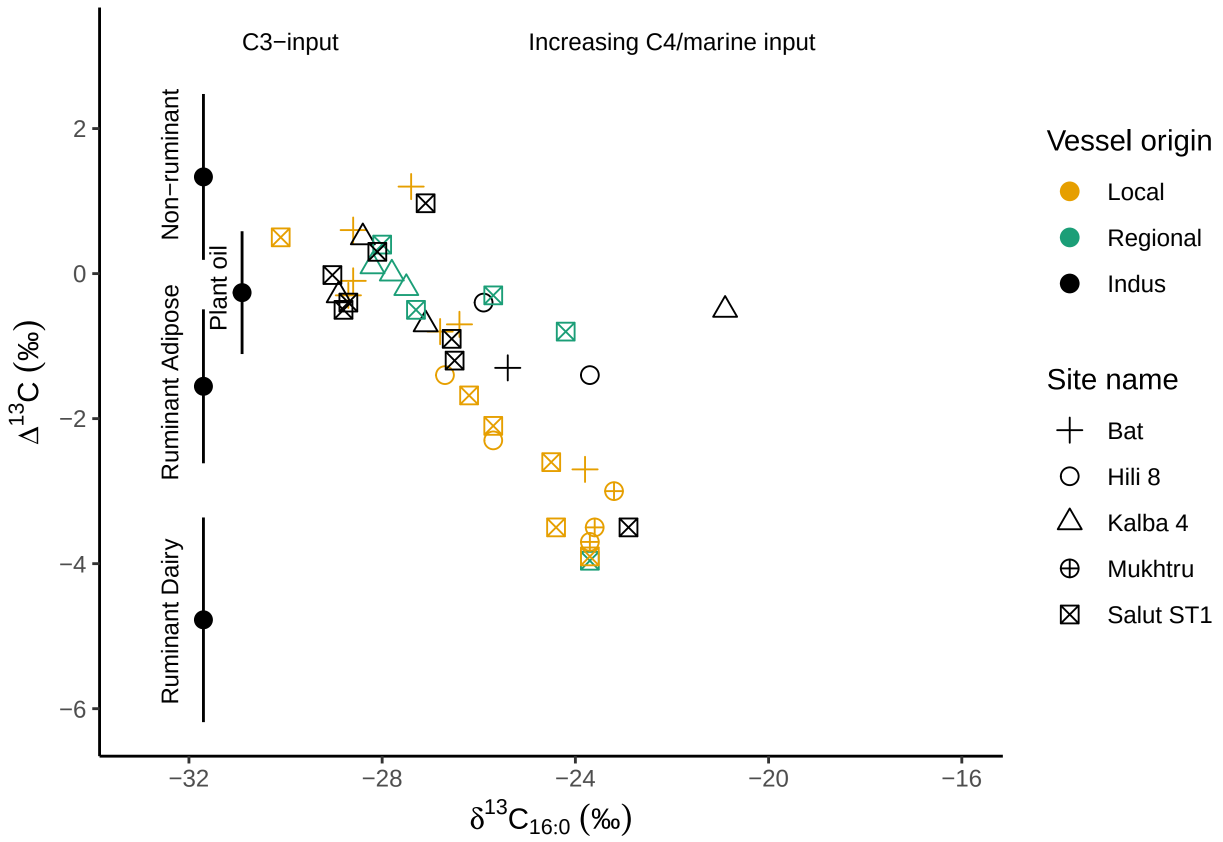

#FIGURE 6

```
f <- ggplot(data = iso_uan, aes(x = d13C16, y = bigdelta)) +
  geom_point(size = 2.8) +
  theme(plot.title = element_text(hjust = 0, size = 16)) +
  expand_limits(x = c(-16, -33), y = c(-6, 3)) +
  scale_y_continuous(breaks = c(-6, -4, -2, 0, 2, 4)) +
  scale_x_continuous(breaks = c(-32, -28, -24, -20, -16)) +
  labs(x = expression(delta^13 * C[16:0] * " "("\u2030")),
       y = expression(Delta^13 * C * " "("\u2030")), colour = "Vessel origin") +
  theme_classic() + facet_wrap(~Site_name)
f
```

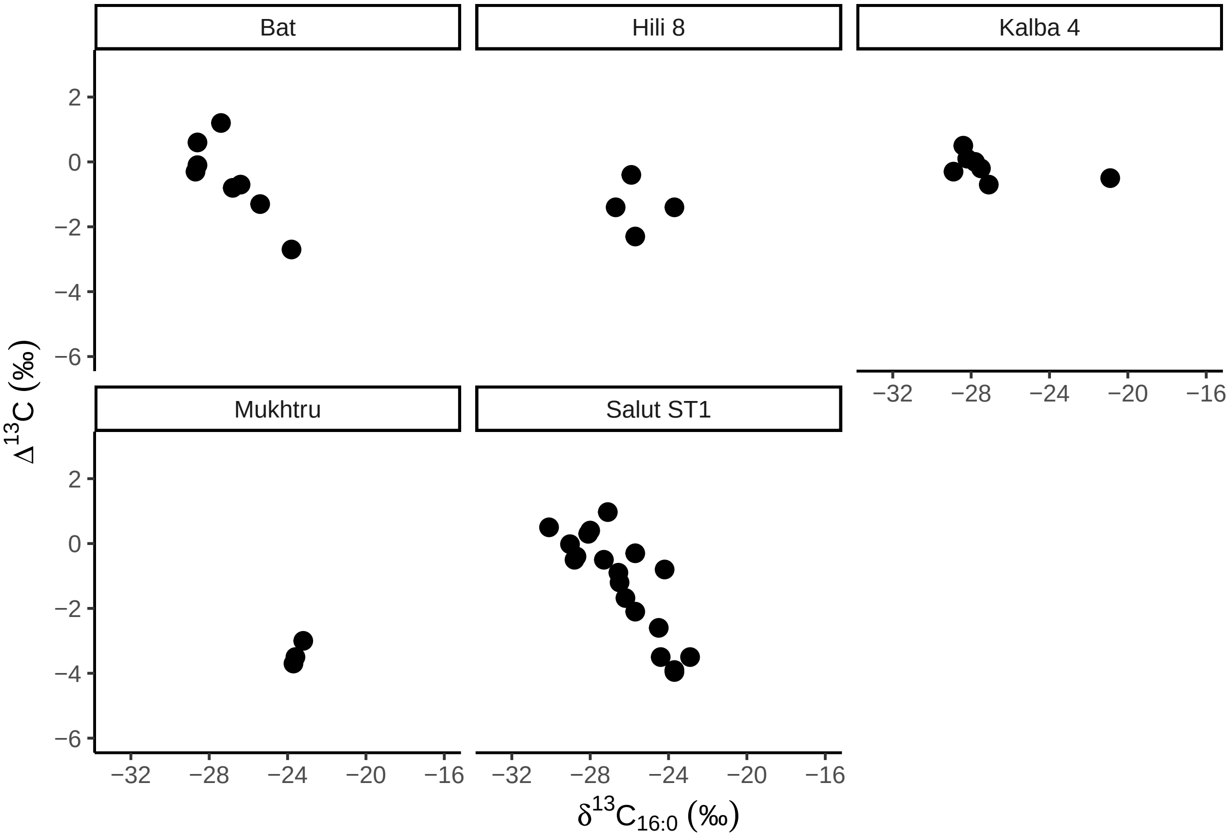


```
# Add the x column to ref.summary2
ref.summary2$x <- rep(c(-30.9, -31.7, -31.7, -31.7), length.out = nrow(ref.summary2))

fig6 <- f + geom_pointrange(data = ref.summary2, aes(x = x, ymin = bigdelta - sd, ymax = bigdelta + sd)
  annotate("text", x = -32.4, y = -4.8, label = "Ruminant Dairy", size = 3.2, angle = 90) +
  annotate("text", x = -32.4, y = -1.5, label = "Ruminant Adipose", size = 3.2, angle = 90) +
  annotate("text", x = -32.4, y = 1.5, label = "Non-ruminant", size = 3.2, angle = 90) +
  annotate("text", x = -31.4, y = -0.2, label = "Plant oil", size = 3.2, angle = 90) +
  annotate("text", x = -29.9, y = 3.2, label = "C3-input", size = 3.2, angle = 0) +
  annotate("text", x = -22, y = 3.2, label = "Increasing C4/marine input", size = 3.2, angle = 0)
fig6
```

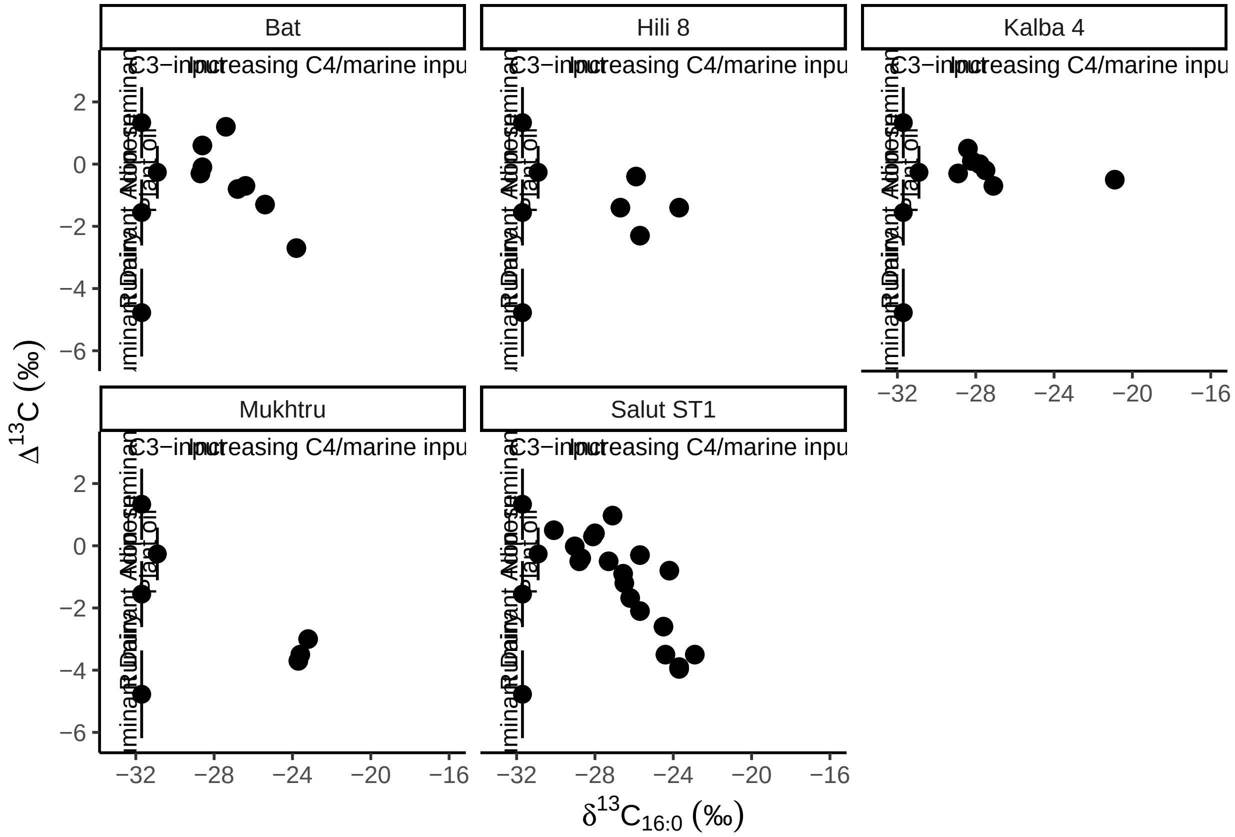
Bat
Hili 8
Kalba 4
Mukhtru
Salut ST1
C3−input
Increasing C4/marine inpu
2
0
−2
−4
−6
−32
−28
−24
−20
−16
Δ13C (‰)
δ13C16:0 (‰)
