# Supplementary material for: Identifying pastoral and plant products in local and imported pottery in Early Bronze Age southeastern Arabia
Source: PLoS One. 2025 Jun 11;20(6):e0324661. doi: 10.1371/journal.pone.0324661 (PMC12157666; doi:10.1371/journal.pone.0324661)
Supplement: S5 File — (CSV) [file pone.0324661.s005.pdf]

S5: Abstract in Arabic

الملخص

إن لأصول تقنية الفخار في شبه الجزيرة العُمانية تاريخًا فريدًا ضمن سياق غرب آسيا القديمة، حيث لم يتم توثيق إنتاج الفخار المحلي في شمال عُمان والإمارات العربية المتحدة إلا في الفترة ما بين بداية إلى منتصف الألفية الثالثة قبل الميلاد، وذلك خلال العصر البرونزي المبكر. وقد تميزت هذه الفترة بزيادة الاستقرار السكني، وتوسّع شبكات التبادل بعيدة المدى التي كانت تعمل عبر الخليج العربي، وربطت بين الجزيرة العربية وبلاد الرافدين وإيران وجنوب آسيا، بما في ذلك تبادل الأواني الفخارية.

ولاستكشاف الروابط بين تقنية الفخار وأنواعه، والممارسات المعيشية، والتحول إلى الاستقرار في الوقت الذي بدأ فيه اعتماد إنتاج الفخار في المنطقة، قمنا بتحليل محتوى الدهون في فخار العصر البرونزي المبكر (بإجمالي 179) وذلك من مواقع داخلية وساحلية في جنوب شرق الجزيرة العربية.

تشمل المجموعة الفخارية التي تمت دراستها أواني صُنعت محليًا على مستوى الموقع، إضافةً إلى أواني تم توزيعها إقليميًا. كما جرت دراسة محتويات الأواني المستوردة من بلاد الرافدين وحضارة السند، والتي تم العثور عليها من مواقع داخلية وساحلية في جنوب شرق الجزيرة العربية، لتحديد المنتجات العضوية التي ربما تم نقلها كجزء من شبكات التبادل بعيدة المدى.

تكشف النتائج عن وجود منتجات رعوية مثل اللحوم ومنتجات الألبان في بعض أقدم الأواني التي صُنعت في جنوب شرق الجزيرة العربية، وكذلك في الأواني المستوردة من بلاد الرافدين. كما تم اكتشاف منتجات نباتية في نسبة قليلة من الأواني سواء محلية الصنع أو المستوردة، مثل الأواني العُمانية الحمراء الناعمة، وجرار التخزين ذات الصبغة السوداء من حضارة السند.

تُظهر هذه الدراسة أهمية استخدام الأساليب الجزيئية البيولوجية في دراسة العادات الغذائية واستخدام الأواني في جنوب شرق الجزيرة العربية على نطاق واسع.

الكلمات المفتاحية : جنوب شرق الجزيرة العربية، العصر البرونزي المبكر، بقايا الدهون، استخدام الأواني، شبكات التبادل
